# Systematic assessment of transcriptomic and metabolic reprogramming by blue light exposure coupled with aging

**DOI:** 10.1093/pnasnexus/pgad390

**Published:** 2023-12-05

**Authors:** Jia Huang, Fan Zhou, Huanchan Zhou, Xiaoqi Zheng, Zhengyi Huo, Meng Yang, Zihe Xu, Runzhou Liu, Luoluo Wang, Xiaoyun Wang

**Affiliations:** Department of Entomology, South China Agricultural University, Guangzhou 510642, China; Guangdong Provincial Key Laboratory of Insect Developmental Biology and Applied Technology, Institute of Insect Science and Technology, School of Life Sciences, South China Normal University, Guangzhou 510631, China; Guangdong Provincial Key Laboratory of Insect Developmental Biology and Applied Technology, Institute of Insect Science and Technology, School of Life Sciences, South China Normal University, Guangzhou 510631, China; Guangdong Provincial Key Laboratory of Insect Developmental Biology and Applied Technology, Institute of Insect Science and Technology, School of Life Sciences, South China Normal University, Guangzhou 510631, China; Guangdong Provincial Key Laboratory of Insect Developmental Biology and Applied Technology, Institute of Insect Science and Technology, School of Life Sciences, South China Normal University, Guangzhou 510631, China; Guangdong Provincial Key Laboratory of Insect Developmental Biology and Applied Technology, Institute of Insect Science and Technology, School of Life Sciences, South China Normal University, Guangzhou 510631, China; Guangdong Provincial Key Laboratory of Insect Developmental Biology and Applied Technology, Institute of Insect Science and Technology, School of Life Sciences, South China Normal University, Guangzhou 510631, China; Guangdong Provincial Key Laboratory of Insect Developmental Biology and Applied Technology, Institute of Insect Science and Technology, School of Life Sciences, South China Normal University, Guangzhou 510631, China; Guangdong Provincial Key Laboratory of Insect Developmental Biology and Applied Technology, Institute of Insect Science and Technology, School of Life Sciences, South China Normal University, Guangzhou 510631, China; Guangdong Provincial Key Laboratory of Insect Developmental Biology and Applied Technology, Institute of Insect Science and Technology, School of Life Sciences, South China Normal University, Guangzhou 510631, China

**Keywords:** RNA methylome, blue light, aging, circadian rhythm

## Abstract

The prevalent use of light-emitting diodes (LEDs) has caused revolutionary changes in modern life, but the potential hazards to health of blue light are poorly understood. N^6^-methyladenosine (m^6^A) is the most prevalent posttranscriptional modification in eukaryotes and can modulate diverse physiological processes by regulating mRNA fate. Here, to understand the effects and molecular mechanisms of daily low-intensity blue light exposure (BLE) and ascertain whether m^6^A methylation plays a role in BLE-induced phenotypes, we constructed a series of *Drosophila* models under different durations of daily low-intensity BLE and obtained multiomics profiles. Our results revealed that BLE could induce transcriptomic, m^6^A epitranscriptomic, and metabolomic reprogramming in *Drosophila* along with aging process. Importantly, the m^6^A methylation sites enriched in the 5′ untranslated regions (UTRs) of *Drosophila* transcripts showed strong age specificity and could be altered by BLE. We experimentally validated that aging-related gene *Tor* and circadian rhythm-related gene *per* were regulated by 5′ UTR-enriched m^6^A methylation. Overall, our study provides a systematic assessment of m^6^A RNA methylome reprogramming by BLE and aging in *Drosophila* model.

**Significance Statement**

The widespread use of light-emitting diodes has caused revolutionary changes to humans. However, the potential hazards in health, cognition, and aging from extensive or long-term exposure to artificial blue light are poorly understood. In this study, we use the model organism *Drosophila melanogaster* to investigate the impacts of blue light exposure with multiomics approaches. Our study provides a systematic assessment of blue light exposure–induced N^6^-methyladenosine epitranscriptomic reprogramming coordinated with aging in an animal model, which will bring attention to the potential hazards of cumulative blue light exposure in humans.

## Background

Natural light is essential for the development of circadian rhythms to coordinate the physiology, metabolism, and behavior of the majority of organisms (1). However, with the rapid development of the lighting and electronics industries in modern society, the amount of human exposure to the high-energy blue light spectrum (wavelengths [λ] = 400–500 nm) derived from artificial lighting, especially light-emitting diodes (LEDs), is increasing (2). Blue light exposure (BLE) has become a potential risk factor for damage accumulation in retinal cells (λ = 400–460 nm; 3–5) and circadian disruption (λ = 460–500 nm; 1, 6). Recent emerging studies focusing on the impacts of daily BLE in animal models have demonstrated that resultant damage can not only affect mental health by causing intrinsically photosensitive retinal ganglion cells to become disordered (6) but can also accelerate aging and cause brain neurodegeneration (7). In addition, high-intensity BLE is associated with the accumulation of reactive oxygen species, which can result in apoptosis and high mortality in the short term (2). However, the influences and molecular mechanisms of daily low-intensity BLE are still poorly understood.

Among the more than 150 known chemical RNA modifications (8), N^6^-methyladenosine (m^6^A) is one of the predominant types and is mainly found on eukaryotic messenger RNAs (mRNAs) and long noncoding RNAs. The development of methylated RNA immunoprecipitation and sequencing (MeRIP-seq) technology has greatly broadened our knowledge of m^6^A epitranscriptomic regulation (9). m^6^A methylation is particularly enriched in the nervous system (10) and is dynamically distributed among thousands of RNA transcripts with unique species-specific, tissue-specific, age-specific, environment-specific, and stimulus-specific patterns (11–14). m^6^A and m^6^A-related factors have been found to play important roles in facilitating mRNA degradation (15), initiating mRNA translation (16), modulating mRNA alternative splicing events (17), transforming RNA structure (18), and promoting pri-miRNA processing (19). m^6^A methylation is catalyzed by a methyltransferase-like 3 and 14-centered multicomponent methyltransferase complex (MTC; referred to as “writers”) and removed by the demethylases fat mass and obesity-associated protein (FTO) and AlkB homolog 5 (ALKBH5; known as “erasers”), conferring the capacity to undergo reversible changes. m^6^A methylation can further be recognized by YTH domain-containing proteins (as “readers”) and modulates mRNA fate in vertebrates (20). Given the recognized role of m^6^A in neuronal functions, an open question is whether the m^6^A epitranscriptome plays a role in blue light-mediated phenotypes including circadian disruption, neurodegeneration, or aging.

*Drosophila* remains one of the widely applied model organisms for carrying out aging-related and circadian rhythm-related studies in vivo (21, 22). Although the identification of m^6^A demethylase in *Drosophila* awaits further investigation (23), recent studies have shown that the MTC and reader proteins involved in the m^6^A module system have been relatively conserved in *Drosophila* during evolution (23–27). In this study, we constructed a series of *Drosophila* models under different durations of daily BLE and investigated their transcriptomic, m^6^A epitranscriptomic, and metabolomic profiles. Overall, our results revealed that BLE had a great impact on adult neuronal functions at the transcriptomic level. Both long-term BLE and aging could trigger directed transcriptomic, m^6^A epitranscriptomic, and metabolomic reprogramming in whole adult flies. m^6^A methylation enriched in the 5′ untranslated regions (UTRs) of transcripts was shown to be the main target of the MTC, showed strong age specificity, and could be altered by BLE. Both aging-related and circadian rhythm-related genes were regulated by 5′ UTR-enriched m^6^A methylation. We propose an underlying mechanism by which aging-induced 5′ UTR-enriched m^6^A epitranscriptomic reprogramming regulates the fate of aging-related mRNAs and further exacerbates the aging phenotype. In addition, both parental age and photoperiod moderately affected the transcriptomic profiles of the F_1_ generation. Collectively, this study provides a systematic assessment of the implications of m^6^A epitranscriptomic reprogramming induced by BLE and aging in *Drosophila*.

## Results

### Transcriptomic profiles reveal potential damage to the cephalic nervous system from BLE

**Fig. 1.**
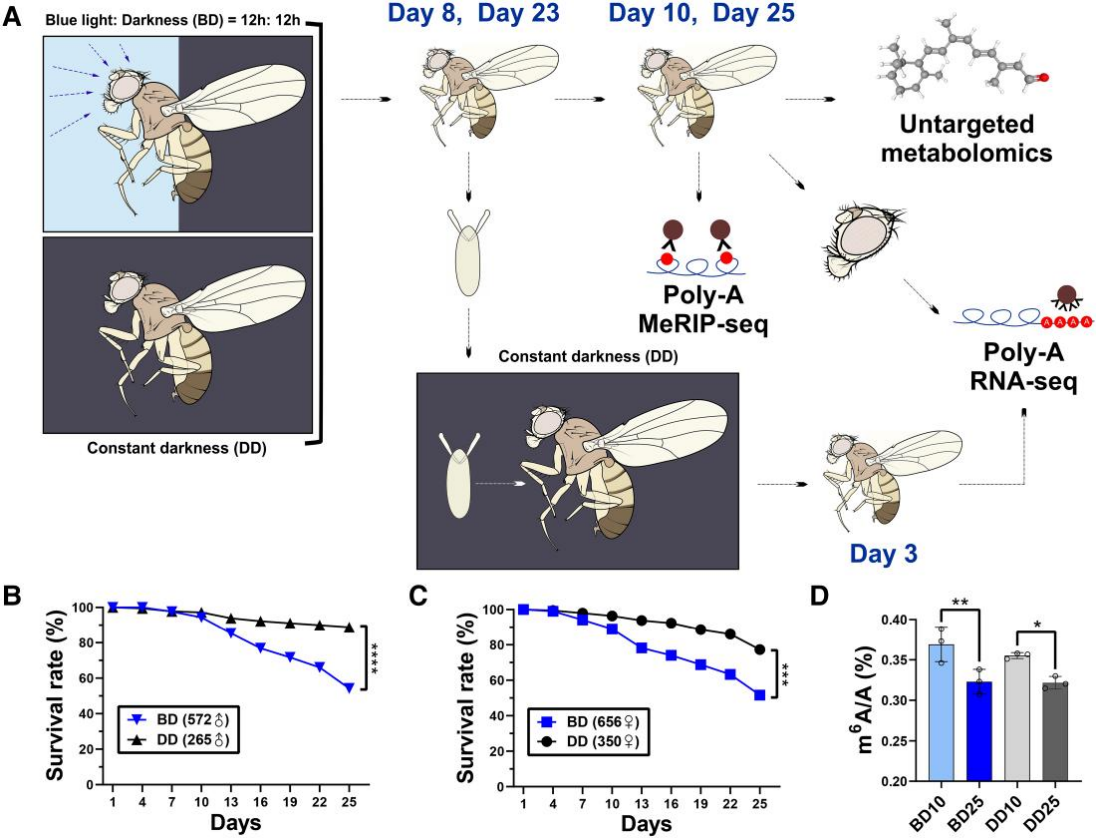
Workflow and preliminary experimental results of this study. A) Schematic illustration of the samples, treatments, and omics techniques used in this study. One day after eclosion, *Drosophila melanogaster w^1118^* fly colonies were reared in a climatic chamber under a photoperiodic cycle of 12 h low-intensity BLE: 12 h darkness (BD) and constant darkness (DD), respectively. To investigate the mRNA profiles of the F_1_ generation under the influence of parental photoperiod and aging, we collected eggs from the 8-day-old and 23-day-old flies, reared them under DD until 3 days after eclosion, and sampled the whole 3-day-old adult male flies for RNA-seq. We then sampled the whole 10-day-old and 25-day-old adult male flies for MeRIP-seq and untargeted metabolome quantification by LC-MS/MS, and we collected adult male heads for RNA-seq. Survival rates of the sampled adult male (B) and female flies (C) under BD compared with DD. ***P < 0.001, ****P < 0.0001, measured with the log-rank test. D) m^6^A levels of poly(A)-RNA quantified by LC-MS/MS. BD10 and BD25: 10-day-old and 25-day-old adult flies reared under the BD photoperiodic cycle, respectively; DD10 and DD25: 10-day-old and 25-day-old adult flies reared under DD, respectively. Mean ± SD; *P < 0.05, **P < 0.01, measured with two-way ANOVA.

To investigate the underlying impacts of daily BLE at the molecular level, we constructed a series of *Drosophila* models and obtained five types of omics data sets (Fig. 1; Table S1). Briefly, we reared 1-day-old adults under a photoperiodic cycle of 12 h low-intensity BLE: 12 h darkness (BD) and constant darkness (DD), respectively. We found the BD flies showed noteworthy motor impairment and a shorter lifespan (Fig. 1B and C). More importantly, quantification by liquid chromatography-tandem mass spectrometry (LC-MS/MS) showed that m^6^A/A level was significantly different for the comparisons between BD10 and BD25 and the comparisons between DD10 and DD25 (Fig. 1D). We then collected whole 10-day-old and 25-day-old adult male flies (BD10, BD25, DD10, and DD25 flies) in bulk for poly(A)-MeRIP-seq (including m^6^A immunoprecipitation [IP] and untreated control [input]) and untargeted metabolome and collected adult male heads (BD10, BD25, DD10, and DD25 heads) in bulk for poly(A)-RNA sequencing (RNA-seq; Fig. 1A; Table S1). To investigate the mRNA profiles of the F_1_ generation under the influence of parental photoperiod and aging, we also collected eggs from the 8-day-old and 23-day-old flies, colonized them under DD until 3 days after eclosion, and sampled these whole 3-day-old adult male flies (BD8F_1_, BD23F_1_, DD8F_1_, and DD23F_1_ flies) in bulk for poly(A)-RNA-seq (Fig. 1A; Table S1).

**Fig. 2.**
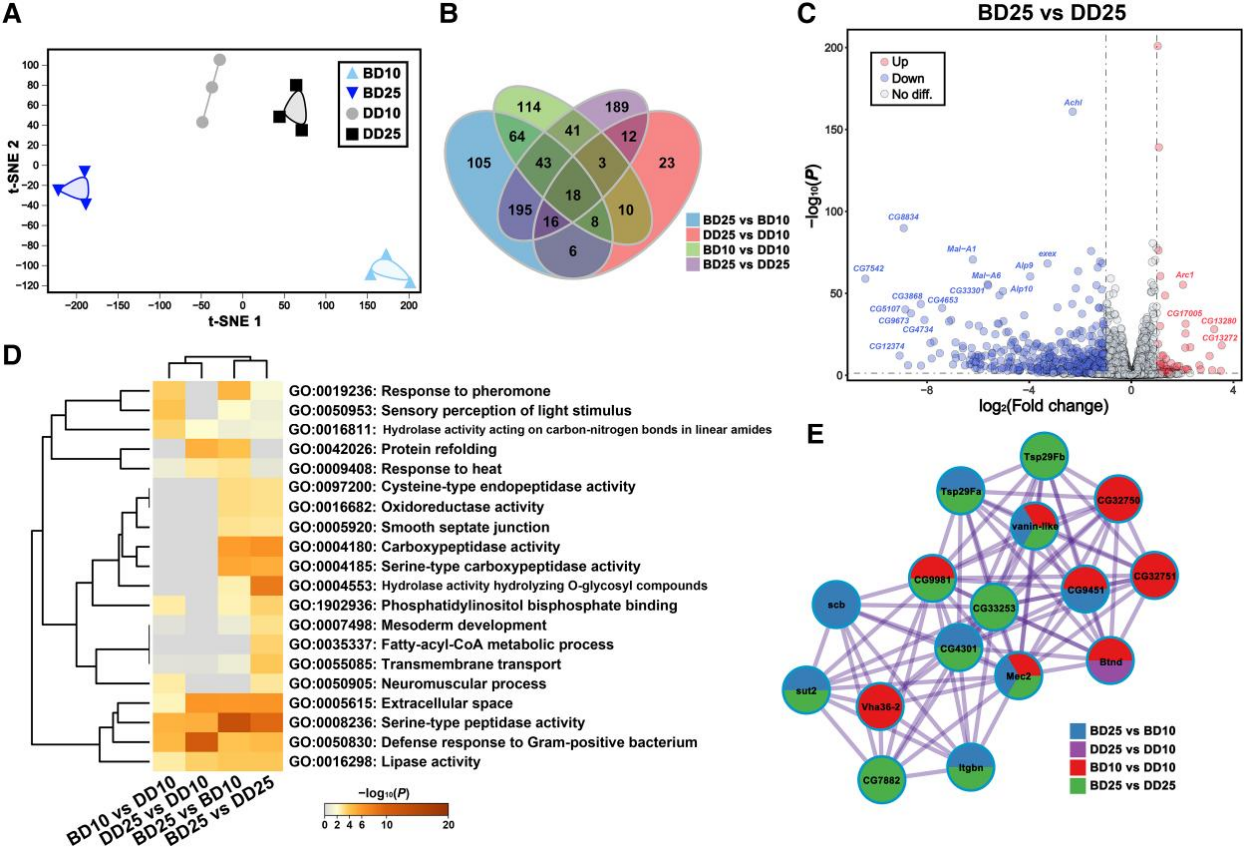
Poly(A)-RNA-seq of adult male heads. A) t-SNE dimension reduction analysis based on 11,350 genes. B) Venn diagram displaying the common and unique DEGs identified in the single-factor comparisons between groups. An FDR-corrected *P*-value <0.05 and fold change ≥2 were used as criteria for identifying DEGs. C) Volcano plot between the BD25 and DD25 heads based on 11,057 genes. *P* < 0.05 and a fold change ≥2 were set as the thresholds of significance. The symbols representing some of the significant DEGs are shown. D) Enrichment and clustering analyses showing the top 20 GO terms of the DEGs identified in the single-factor comparisons between groups. E) The most significant PPI network based on the DEGs identified in the single-factor comparisons between groups.

We started with analysis on the RNA-seq data of adult male heads. Overall, a total of 11,350 genes from the samples were filtered and used for subsequent analyses. t-distributed Stochastic Neighbor Embedding (t-SNE) analysis indicated that all four groups could be distinctly separated from each other based on global gene expression patterns (Fig. 2A). Comparatively, the global RNA profiles were slightly different between the DD10 and DD25 heads but significantly different between the BD10 and BD25 heads (Fig. 2A), suggesting that BLE has a greater influence than aging. Differential expression analyses based on the single-factor comparisons between groups showed 96–517 differentially expressed genes (DEGs) screened out by DESeq2, and the greatest number of DEGs was derived from the comparison between the BD25 and DD25 heads (Fig. 2B; Table S2). It is worth noting that most of the 517 DEGs identified between the BD25 and DD25 heads were down-regulated (Fig. 2C), and according to the annotations in the FlyBase database, most of these significantly down-regulated DEGs were specifically expressed in adult heads and related to physiological metabolism. Extra-extra (*exex*), which encodes a homeodomain transcription factor that plays a role in the specification and differentiation of motor neurons, was one of the significantly down-regulated gene identified between the BD25 and DD25 heads.

Based on the significant enrichment identified between the BD10 and DD10 heads and the BD25 and DD25 heads, Gene Ontology (GO) enrichment analysis indicated that the neuromuscular process term (GO: 0050905) was associated with BLE (Fig. 2D). The proteins encoded by 16 DEGs constituted the most significant protein–protein interaction (PPI) network (Fig. 2E). Among these proteins, the human orthologs of sugar transporter 2 (sut2) and CG7882 are implicated in multiple brain diseases, integrin beta-nu subunit (Itgbn) is related to neuromuscular junction growth, and tetraspanin 29Fb (Tsp29Fb) is associated with nonsyndromic X-linked intellectual disability. Gene set enrichment analyses (GSEAs) between the BD25 and DD25 heads demonstrated that long-term BLE could induce the universal up-regulation of neurotransmitter transport-related genes (Fig. S1A) and the down-regulation of nervous system development-related genes (Fig. S1B). Altogether, these results revealed the potential damage induced by long-term daily BLE in the neuronal functions of *Drosophila* at the transcriptomic level.

### BLE and aging induce transcriptomic reprogramming in adult flies

**Fig. 3.**
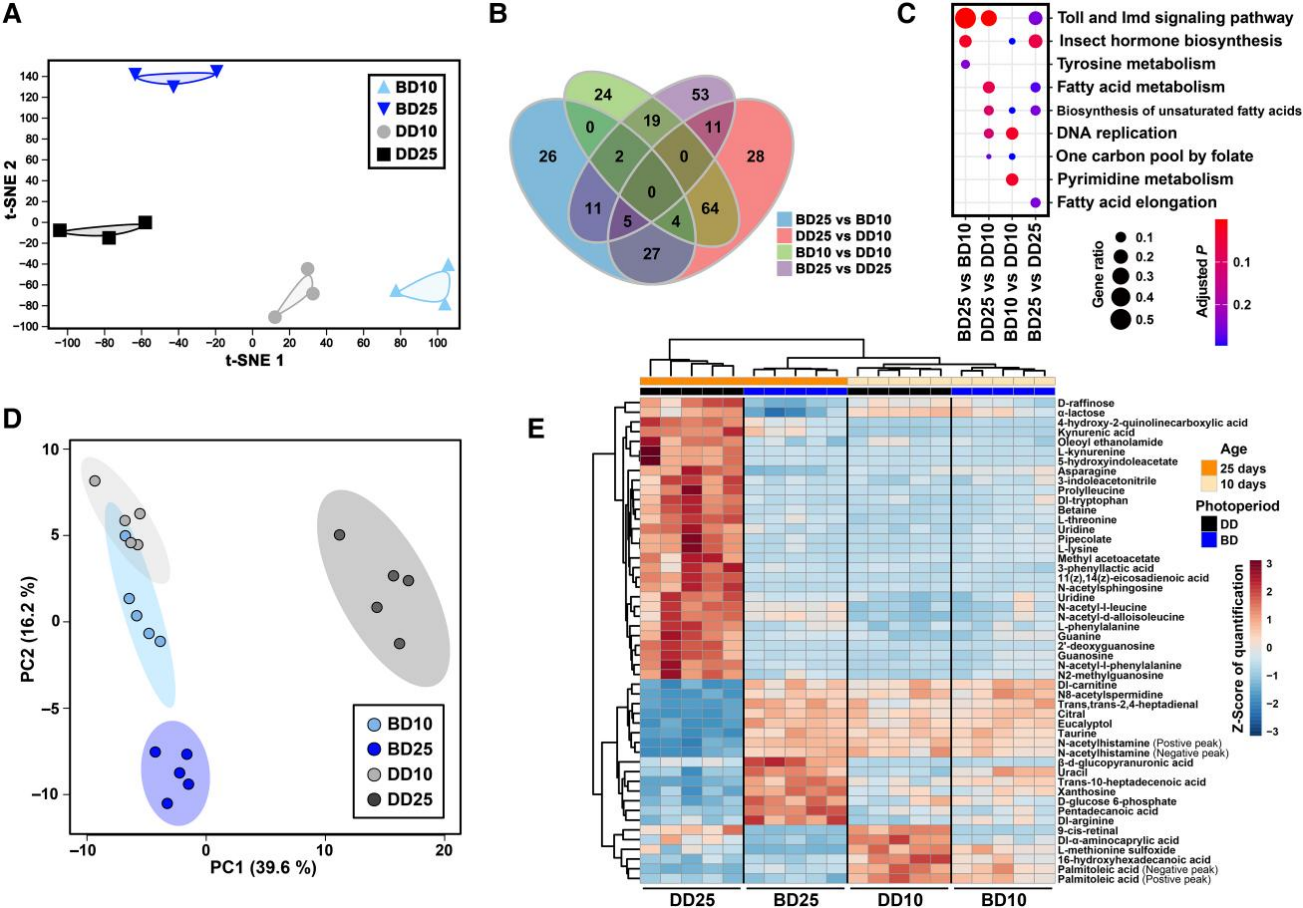
Poly(A)-RNA-seq and untargeted metabolomic analysis of whole adult male flies. A) t-SNE dimension reduction analysis based on 13,636 genes. B) Venn diagram displaying the common and unique DEGs identified in the single-factor comparisons between groups. An FDR < 0.05 and a fold change ≥2 were used as criteria for identifying DEGs. C) KEGG enrichment analysis based on the DEGs identified in the single-factor comparisons between groups. D) PCA based on 208 quantified compounds. E) Heatmap and clustering analysis of the top 50 compounds with high levels.

According to the same pipeline, the global RNA profiles of whole flies were evaluated based on the input data of MeRIP-seq and compared with the head RNA-seq data. Similar to the results obtained from the heads, all four groups remained at a considerable distance from each other in the t-SNE analysis based on 13,636 genes of whole flies, but the difference between the DD10 and DD25 flies was significantly greater than the differences between the other groups (Fig. 3A). Compared with the results of the other three groups, the cumulative curve of the DD25 flies was slightly deviated due to the reduced proportion of highly expressed genes (Fig. S2A). We also investigated the expression of m^6^A-related and *N*^6^,2′-O-dimethyladenosine (m^6^Am)-related genes (Fig. S2B). As the main players in the MTC, *Mettl3* and *Mettl14* were relatively highly expressed in the BD10 and DD10 flies (Fig. S2B), which was consistent with the quantification of m^6^A levels by LC-MS/MS (Fig. 1D).

Differential expression analyses identified 75–139 DEGs in the single-factor comparisons between groups, and the maximum number of DEGs was identified from the comparison between the DD25 and DD10 flies (Fig. 3B; Table S2), indicating that aging might have a greater impact on the global transcriptomic profiles of whole flies. In the comparison between the BD25 and DD25 groups, the heads and whole flies shared only a small portion of consistently up-regulated and down-regulated genes (Fig. S2C). Most of the DEGs tended to be significantly down-regulated in the heads rather than in the whole flies (Fig. 2C; Fig. S2D). Therefore, the transcriptomic profiles showed a relatively strong tissue-specific pattern, which were consistent with the previous studies showing that eye/brain tissues of *Drosophila* were the major tissues affected by blue light (2, 28–30).

Among the top 20 significantly enriched GO terms, no neurology-related terms were found (Fig. S2E). Instead, defense response-related terms (GO: 0050832 and 0050830) were significantly enriched between the BD25 and BD10 flies and between the DD25 and DD10 flies (Fig. S2E). Kyoto Encyclopedia of Genes and Genomes (KEGG) enrichment analysis showed that the DEGs were closely related to the Toll and Imd signaling pathways (KEGG: dme04624; Fig. 3C), which play important roles in autophagy, apoptosis, and defense responses in *Drosophila*. Therefore, both BLE and aging could trigger directed transcriptomic reprogramming in whole adult flies.

### BLE and aging cause metabolomic reprogramming in adult flies

To provide more information on the correlation between transcription and metabolism, untargeted metabolomic quantification was performed on the same fly samples subjected to MeRIP-seq in this study. We assessed the quantification results of 208 compounds that were identified with high confidence levels in this study by both principal component analysis (PCA) (Fig. 3D) and t-SNE analysis (Fig. S3A). The PCA results, in which PC1 and PC2 contributed 55.8% of the total variance, showed that the DD25 and BD25 flies could both be separated from the other groups, although the 10-day-old flies showed partial overlap (Fig. 3D). The t-SNE analysis supported the absence of outliers between the BD10 and DD10 flies (Fig. S3A). Both of the dimensional reduction analyses indicated that DD25 was the group with the maximum compound differences relative to the other three groups (Fig. 3D; Fig. S3A). In addition, more than half of the compounds showed high levels specifically in the DD25 flies (Fig. 3E). In short, the patterns of the global transcriptomic (Fig. 3A) and metabolomic profiles of whole adult flies (Fig. 3D; Fig. S3A) were generally similar.

We identified 3–63 differential compounds (Fig. S3B; Table S2) according to quantification with Student's t test. It is worth noting that one of the differential compounds, 9-cis-retinal (Fig. S3B), showed a significant reduction in the BD flies (BD versus DD, *P* < 0.0001; Fig. 3E). It has been demonstrated that 9-cis-retinal is highly consistent with the degenerated retinal photoreceptor phenotype caused by BLE in *Drosophila* (2).

Based on the identified differential compounds, aspartate metabolism and carnitine synthesis were significantly enriched between the BD25 and DD25 flies (Fig. S3C) and the DD25 and DD10 flies (Fig. S3D), respectively. Taking L-glutamine as an example, we noted that glutaminase (GLS), a hydrolase that catalyzes the transformation of this compound to L-glutamate (KEGG: dme00250), showed some increase in *GLS* 5′ UTR-enriched m^6^A methylation in the BD25 flies relative to the DD25 flies based on the MeRIP-seq data. Collectively, the results indicated that the metabolome of whole adult flies could also be reprogrammed by both BLE and aging. In addition, our results indicated a certain degree of consistency between the transcriptome and metabolome and an association between the m^6^A epitranscriptome and metabolome.

### Epitranscriptomic profiles show that 5′ UTR-enriched m^6^A methylation presents strong age specificity and can be altered by BLE

**Fig. 4.**
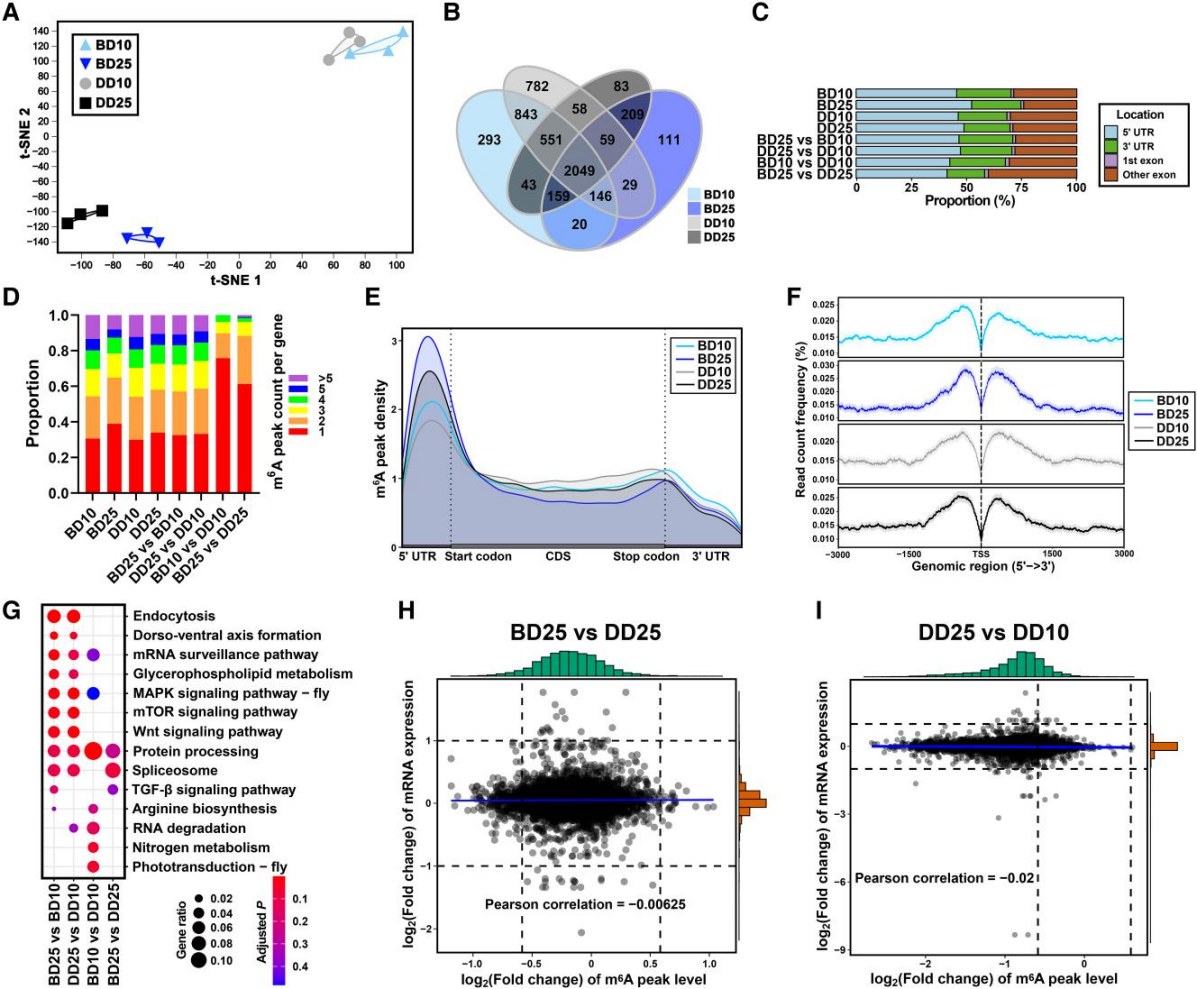
MeRIP-seq of whole adult male flies. A) t-SNE dimension reduction analysis based on 12,619 genes. B) Venn diagram displaying the common and unique methylated genes among groups. C) Annotation locations in genes of the significant (differential) m^6^A peaks. D) Proportions of the m^6^A methylated and DMGs with different m^6^A peak counts. E) m^6^A peak distribution across the mRNA meta-transcript, showing differences in enrichment around annotated locations. F) m^6^A read count frequency near TSSs. G) KEGG enrichment analysis based on the m^6^A DMGs identified in the single-factor comparisons between groups. Nine-quadrant diagrams showing that the m^6^A epitranscriptome and transcriptome are weakly negatively correlated (H) between the BD25 and DD25 flies and (I) between the DD25 and DD10 flies. A fold change ≥2 (1.5), *P* < 0.01, and an FDR < 0.01 were used as criteria for identifying significant (differential) m^6^A peaks.

The global m^6^A methylation profiles of whole adult male flies were further assessed based on the MeRIP-seq IP data. t-SNE analyses were performed at both the gene level (based on 12,618 genes; Fig. 4A) and the peak level (based on 6,332 consistent peaks; Fig. S4A) among the fly samples. The two analyses showed similar results indicating that the m^6^A profiles showed strong age specificity and could be slightly altered under long-term BLE. The numbers of consistent significant m^6^A peaks identified in the BD10, BD25, DD10, and DD25 flies were 4,103, 2,781, 4,516, and 3,210, respectively (Fig. 4B; Table S3). Considered together with the quantification of m^6^A levels by LC-MS/MS (Fig. 1D), the results of the two analyses revealed a consensus that aging led to a decrease at the m^6^A level, whereas they diverged regarding the influence of BLE on m^6^A methylation.

We identified 516–7,166 significant differential m^6^A peaks belonging to 443–4,027 differentially methylated genes (DMGs) in the single-factor comparisons between groups (Fig. S4B; Table S3). The annotation results showed that the significant m^6^A peaks of the 25-day-old flies tended to be enriched in the 5′ UTRs of transcripts (Fig. 4C). In addition, the average m^6^A peak number among methylated genes was reduced in the 25-day-old flies (Fig. 4D). The peak distribution characteristics across the mRNA displayed similar results: significant m^6^A peaks were significantly enriched in the 5′ UTRs of transcripts, especially in the 25-day-old flies (Fig. 4E). On the other hand, the BD flies showed comparatively higher 5′ UTR-enriched m^6^A methylation (Fig. 4E). Measured based on peak scores, the significant m^6^A peaks of the 25-day-old flies also presented lower enrichment levels relative to the 10-day-old flies (Fig. S4C). In addition, we found that the summits of the significant m^6^A peaks were located 400–600 bp away from transcriptional start sites (TSSs; Fig. 4F); thus, 5′ UTR-enriched m^6^A methylation was cap independent in *Drosophila*. Furthermore, an “ACAACA” motif was calculated as one of the most frequent and significant motifs in all four groups (Fig. S4D).

We next performed KEGG enrichment analysis and found that a series of KEGG terms, including the mechanistic target of rapamycin (mTOR) signaling pathway (KEGG: dme04150), were significantly enriched between the BD25 and BD10 flies and between the DD25 and DD10 flies (Fig. 4G), implying that the m^6^A levels of the genes in this pathway were closely related to the aging phenotype of *Drosophila*. Correlation analyses between the global transcriptomic and m^6^A epitranscriptomic profiles indicated that both BLE (Fig. 4H) and aging (Fig. 4I) could shift their linear relationship from the expected positively correlated relationship to being weakly negatively correlated, implying the presence of an inhibitory effect between the global transcriptome and m^6^A epitranscriptome under the impact of either factor.

**Fig. 5.**
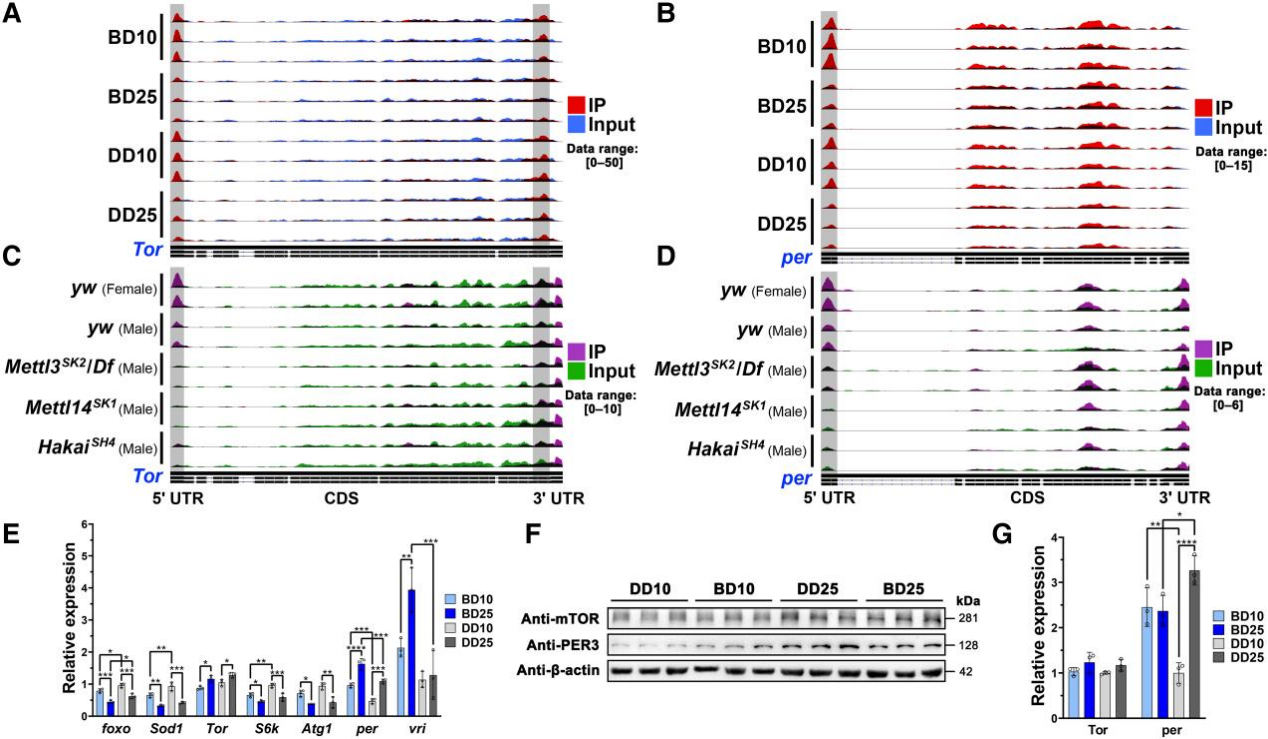
Differential m^6^A enrichment in the 5′ UTRs with RT-qPCR and Western blot validation. IGV tracks displaying the differential read coverage across (A) *Tor* and (B) *per* according to the MeRIP-seq IP and input data of whole adult male flies and (C) *Tor* and (D) *per* according to the MeRIP-seq IP and input data of the *yw* and MTC RNA interference (RNAi) strains (26). The data ranges of all the IGV tracks in each panel have been scaled to the same level. E) RT-qPCR results of aging-related and circadian rhythm-related mRNAs in whole adult male flies. F) Western blotting of Tor and per in whole adult male flies. G) Quantification of relative Tor and per protein expression levels based on the integrated density values (IDVs) from the Western blot images in (F). Mean ± SD; *P < 0.05, **P < 0.01, ***P < 0.001, ****P < 0.0001, measured with two-way ANOVA.

A series of widely known aging-related genes in *Drosophila* were subsequently selected to investigate their mapping coverage according to both the MeRIP-seq IP and input data based on Integrative Genomics Viewer (IGV) tracks, including autophagy-related 1 (*Atg1*), forkhead box, subgroup O (*foxo*), ribosomal protein S6 kinase (*S6k*), superoxide dismutases 1 to 3 (*Sod1*, *Sod2*, and *Sod3*), and the mTOR (*Tor*). *Tor*, which is the human ortholog of *mTOR*, was taken as an example for visualization (Fig. 5A) since the down-regulation of the mTOR nutrient-sensing signaling network can extend lifespan and improve health during aging in diverse organisms (31). It is worth noting that the m^6^A IP reads were mainly enriched in the 5′ UTRs and near the 3′ UTRs of *Tor* with high age specificity (Fig. 5A). Compared with the 25-day-old flies, the 10-day-old flies showed more significant m^6^A peaks in the 5′ UTRs (Fig. 5A). On the other hand, m^6^A enrichment near the 3′ UTRs was sensitive to long-term BLE because of the reduced m^6^A enrichment near the 3′ UTRs in the BD25 flies (Fig. 5A). Similar to the *Tor* results, we found significant age-dependent differential m^6^A peaks in the 5′ UTRs of *Atg1*, *foxo*, and *S6k*. Another interesting phenomenon was that m^6^A enrichment near the 3′ UTRs of *Sod2* was markedly altered between the 10-day-old and 25-day-old flies (Fig. S5A). The results of transcript quantification showed that the expression of the two transcripts (*Sod2-RA* and *Sod2-RB*) was negatively correlated between the IP and input data (Fig. S5B).

Blue light may also play a crucial role in circadian rhythm entrainment. Therefore, we also checked the IGV tracks of circadian rhythm-related genes in the KEGG pathway (dme04711). As another example, the expression of the period (*per*), which also showed 5′ UTR-enriched m^6^A methylation (Fig. 5B), could be altered not only by age but also by BLE, as the enrichment levels of the BD25 flies were decreased relative to those of the DD25 flies. Similarly, shaggy (*sgg*) and vrille (*vri*) genes, whose products are closely related to circadian rhythms, were also identified as significant DMGs between the BD25 and DD25 flies (Table S3). Altogether, our epitranscriptomic results showed that 5′ UTR-enriched m^6^A methylation presented high age specificity and that its enrichment in neuronal functions-related transcripts could be altered by BLE.

### 5′ UTR-enriched m^6^A methylation is involved in the transcriptional regulation of aging-related and circadian rhythm-related genes

We investigated the binding and active sites of the MTC in the genes of interest using the MeRIP-seq data of the *yw* and in a previously published study (26). The IGV tracks of *Tor* and *per* showed that 5′ UTR-enriched m^6^A methylation was significantly lower in the *Mettl3^SK2^/Df*, *Mettl14^SK1^*, and *Hakai^SH4^* flies than in the *yw* flies, whereas 3′ UTR-enriched m^6^A methylation was insensitive to MTC RNAi (Fig. 5C and D). Similar results were found for *Atg1*, *foxo*, *S6k*, *sgg*, and *vri*. This strong evidence indicated that 5′ UTR-enriched m^6^A methylation was not only present in the targeted binding and active sites of the MTC but was also alterable under specific physiological conditions.

We performed RT-qPCR for a series of genes of interest to validate their relative mRNA expression levels in whole adult male flies. Overall, the 10-day-old flies generally presented higher relative expression levels of m^6^A-related mRNAs (*Mettl3*, *Mettl14*, *fl(2)d*, *Ythdc1*, and *Ythdf*; Fig. S5C), and the 25-day-old flies generally presented higher relative expression levels of potential aging biomarkers (*AttA*, *AttB*, *Def*, *DptA*, *DptB*, *Dro*, and *Drs*; Fig. S5D). Interestingly, short-term BLE significantly reduced the relative expression levels of all of these biomarker genes between the BD10 and DD10 flies (Fig. S5D). With the exception of *Tor*, the other aging-related genes (*foxo*, *Sod1*, *S6k*, and *Atg1*) displayed higher expression in the 10-day-old flies (Fig. 5E). In addition, BLE significantly increased the relative expression of circadian rhythm-related genes in the BD flies (Fig. 5E). The BD25 flies exhibited relatively higher mRNA expression and lower 5′ UTR-enriched m^6^A methylation of *Tor* and *per* (Fig. 5A and B), suggesting that m^6^A might suppress the mRNA levels of these genes.

The relative protein expression levels of Tor and per were determined in the same whole adult male flies by Western blotting. The relative quantification results of the mRNA and protein levels were consistent for *Tor* (Fig. 5E–G), supporting the important role of 5′ UTR-enriched m^6^A methylation in suppressing mRNA levels or inhibiting translation. The relative quantification results of mRNAs and proteins were not completely consistent, but it was still clear that the reduction in 5′ UTR-enriched m^6^A methylation in the 25-day-old flies contributed to the relatively higher expression levels of per (Fig. 5E–G). Taken together, the results indicated that the protein expression levels observed in *Drosophila* were affected by mRNA expression levels, 5′ UTR-enriched m^6^A methylation, and the interaction thereof.

**Fig. 6.**
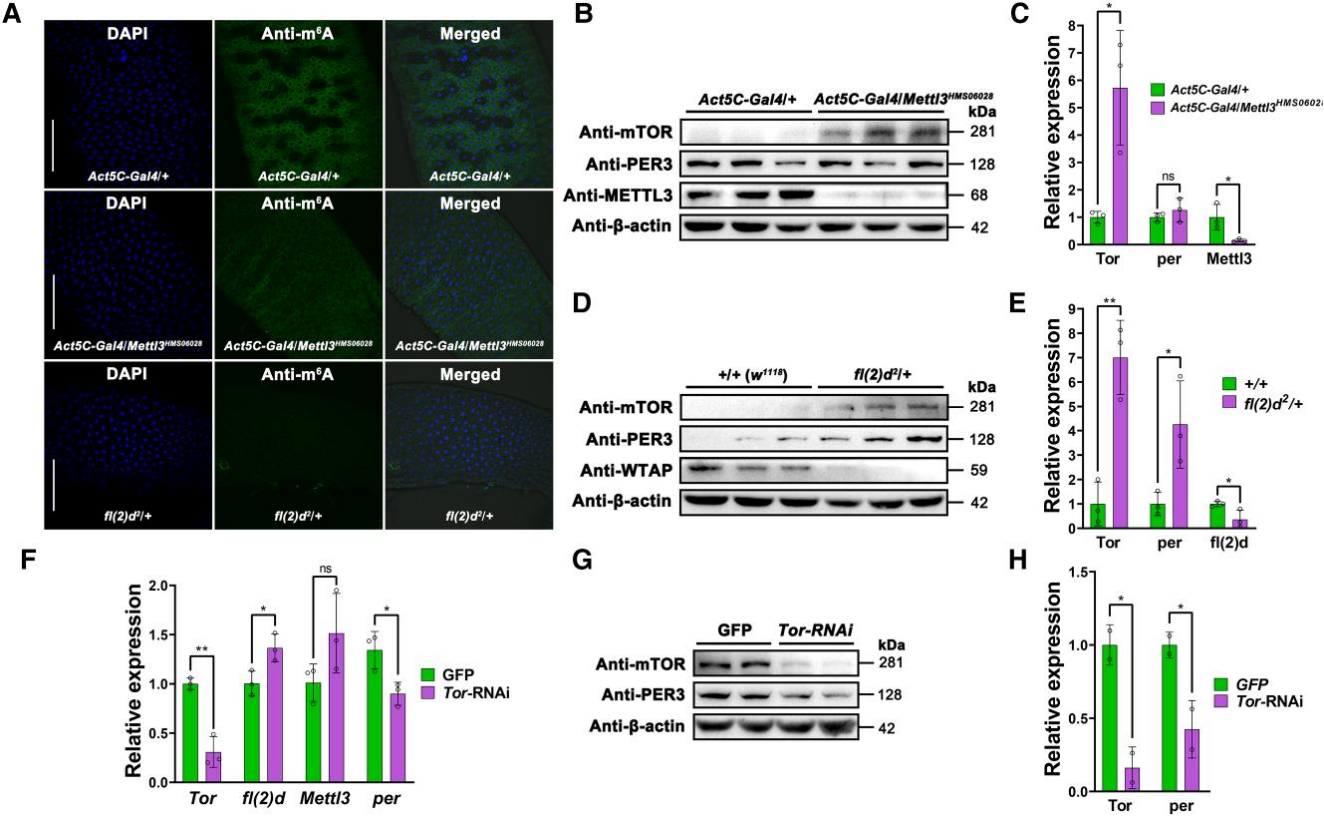
Experimental validations in *Drosophila* strains and *Tor*-RNAi S2 cells. A) Confocal immunofluorescence visualization of midguts of adults from the *Act5C-Gal4*/*+*, *Act5C-Gal4*/*Mettl3^HMS06028^*, and *fl(2)d^2^*/*+* strains fluorescently stained with DAPI (left panels) and the m^6^A primary antibody (middle panels) and corresponding merged images (right panels). B) Western blotting of Tor, per, and Mettl3 in the *Act5C-Gal4*/*+* and *Act5C-Gal4*/*Mettl3^HMS06028^* whole adult male flies. C) Quantification of relative Tor, per, and Mettl3 protein expression levels based on the IDVs from the Western blot images in (B). D) Western blotting of Tor, per, and fl(2)d in the wild-type control *w^1118^* (*+*/*+*) and *fl(2)d^2^*/*+* whole adult male flies. E) Quantification of relative Tor, per, and fl(2)d protein expression levels based on the IDVs from the Western blot images in (D). F) RT-qPCR results of the *Tor*, *fl(2)d*, *Mettl3*, and *per* in the green fluorescent protein (GFP) control and *Tor*-RNAi S2 cells. G) Western blotting of Tor and per in the GFP control and Tor-RNAi S2 cells. H) Quantification of relative Tor and per protein expression levels based on the IDVs from the Western blot images in (G). Mean ± SD; *P < 0.05, **P < 0.01, measured with the unpaired, two-tailed Student's t test.

To further investigate the impacts of the alterations of 5′ UTR-enriched m^6^A methylation levels induced by aging, BLE, or other factors, we constructed recombinant *Mettl3*- and *fl(2)d*-RNAi *Drosophila* strains (for in vivo analysis) and S2 cell lines (for in vitro analysis). Confocal immunofluorescence visualization showed that m^6^A levels were significantly reduced in the midguts of adults of the two recombinant strains (*Act5C-Gal4*/*Mettl3^HMS06028^* and *fl(2)d^2^*/*+*) (Fig. 6A), indicating that the target strains had been successfully constructed and could be used for further experimental validation. Compared with the respective control groups, the relative protein expression levels of Mettl3 or fl(2)d were significantly decreased in the *Act5C-Gal4*/*Mettl3^HMS06028^* and *fl(2)d^2^*/*+* flies, whereas the levels of both Tor and per were generally increased (Fig. 6B–E). In addition, we obtained similar results from *Mettl3*- and *fl(2)d*-RNAi S2 cells (Fig. S6). These results further demonstrated the negative regulation of protein expression by mRNA m^6^A methylation. Another interesting finding was derived from the *Tor*-RNAi S2 cells. RT-qPCR showed successful suppression of *Tor*, together with the up-regulation of *Mettl3* and *fl(2)d* and the significant down-regulation of *per* (Fig. 6F). We obtained consistent results regarding the relative protein expression levels of Tor and per (Fig. 6G and H).

**Fig. 7.**
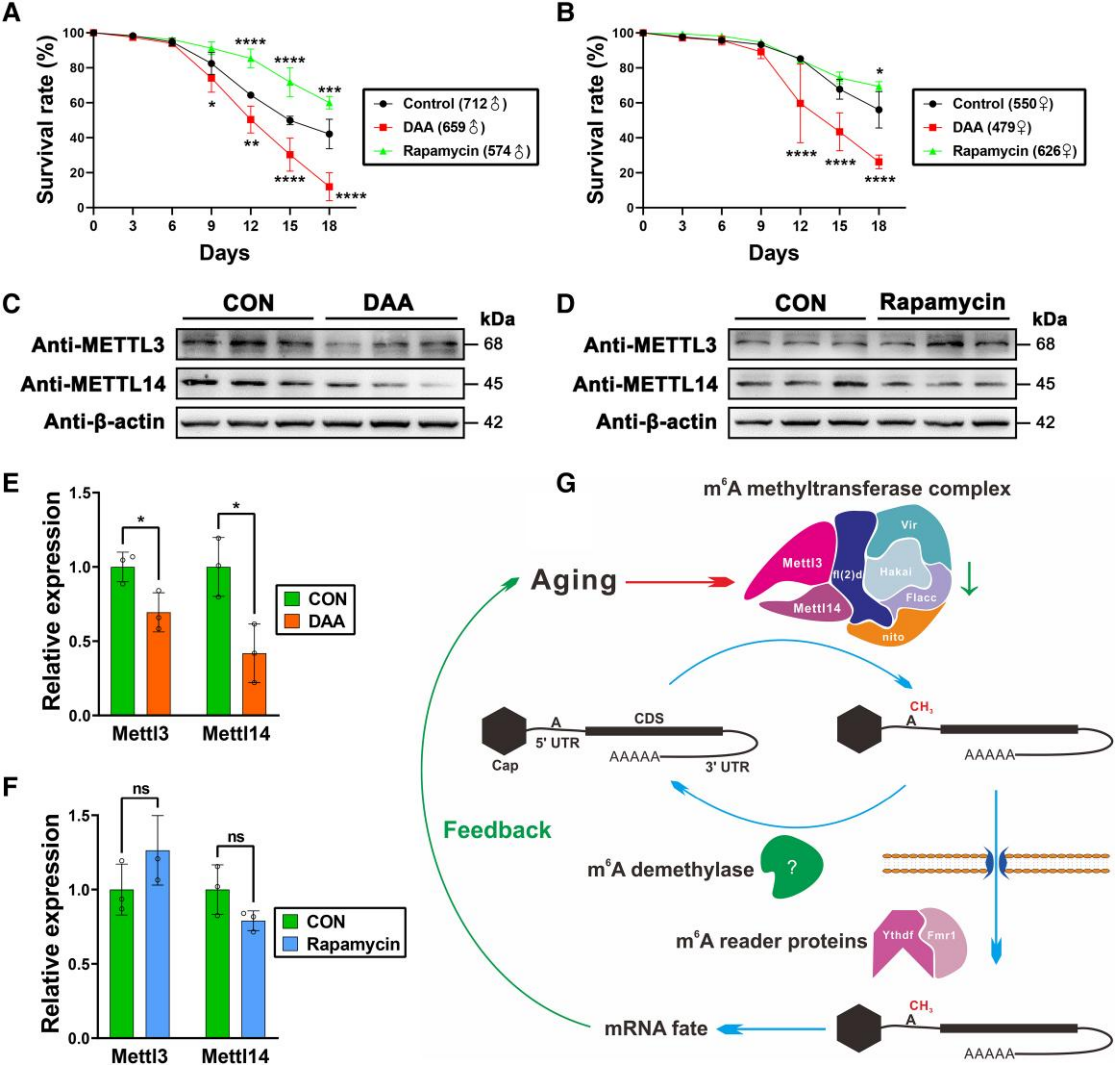
Rescue experiments and proposed working model in this study. A) Survival rates of male flies under blue light treated with methylation inhibitor DAA or mTOR inhibitor rapamycin, compared with untreated control (*n* = 3 each). B) Survival rates of female flies under blue light treated with DAA or rapamycin, compared with untreated control (*n* = 3 each). C) Western blotting of METTL3/14 in adult flies under blue light after DAA treatment, compared with untreated control (*n* = 3 each). D) Western blotting of METTL3/14 in adult flies under blue light after rapamycin treatment, compared with untreated control (*n* = 3 each). E) Quantification of relative METTL3/14 expression levels in DAA and the control, respectively. F) Quantification of relative METTL3/14 expression levels in rapamycin and the control, respectively. G) Proposed working model in this study. Our results from this study suggest that aging-induced 5′ UTR-enriched m^6^A epitranscriptomic reprogramming could regulate the fate of aging-related mRNAs, which could further exacerbate the aging processes as a feedback response. Mean ± SD; *P < 0.05, **P < 0.01, ***P < 0.001, ****P < 0.0001; (A) and (B) measured with two-way ANOVA; (E) and (F) measured with the unpaired, two-tailed Student's t test.

To verify the regulation of m^6^A modification and *Tor* in blue light-induced phenotypes, we performed rescue experiments using methylation inhibitor 3-deazaadenosine (DAA) and mTOR inhibitor rapamycin, respectively. The results showed that DAA can significantly decrease the survival rate for both male (Fig. 7A) and female flies (Fig. 7B), and rapamycin can significantly increase the survival rate for both male (Fig. 7A) and female flies (Fig. 7B). We also showed that DAA significantly reduced the expression of METTL3/14 (Fig. 7C and E), which suggested that DAA can decrease the survival rate in an m^6^A-dependent manner. While rapamycin can increase the survival rate, the expression of METTL3/14 was not obviously changed (Fig. 7D and F), suggesting that the *Tor*-regulated survival rate was m^6^A independent. Overall, our results indicated that blue light-induced phenotypes can be changed by either methylation inhibitor or mTOR inhibitor.

Based on our results, there was a clear association among m^6^A-related genes, 5′ UTR-enriched m^6^A epitranscriptomic regulation, aging-related genes, and the aging phenotype in *Drosophila*. Considering the known m^6^A mechanisms of *Drosophila* (10, 24, 25, 27), we proposed an underlying mechanism. Reduction of 5′ UTR-enriched mRNA m^6^A methylation levels induced by aging coupled with other factors (e.g. blue light, etc.) could alter the fate of aging-related genes, which could further exacerbate the aging processes as a feedback response (Fig. 7G).

### Parental age and photoperiod influence the offspring transcriptome

Since we observed dramatic transcriptional changes of parental *Drosophila* induced by blue light, we were curious about the potential influences of parental age and photoperiod on offspring, so we also analyzed the poly(A)-RNA-seq data of the whole adult male flies from F_1_ generation. With the exception of DD8F_1_ flies, the other three groups could be distinctly separated from each other in the t-SNE analysis based on 13,848 genes (Fig. S7A). Satisfactory results were obtained in another t-SNE analysis (Fig. S7B) based on a total of 211 DEGs derived from the 24–178 DEGs identified in the single-factor comparisons between groups (Fig. S7C; Table S2) after removing replicates. In this analysis, every group maintained a considerable distance from each other, and the difference between the DD8F_1_ and DD23F_1_ flies was significantly greater than that between the BD8F_1_ and BD23F_1_ flies (Fig. S7B). The heatmap displaying the expression levels of these 211 DEGs provided an explanation for the markedly different numbers of DEGs between groups, as certain DEGs were relatively highly expressed in the DD8F_1_ flies (Fig. S7D).

GO and KEGG analyses between the DD8F_1_ and DD23F_1_ flies showed the enrichment of more related terms, including the defense response to Gram-positive bacterium (GO: 0050830) in the GO analysis (Fig. S7E) and the Toll and Imd signaling pathways in the KEGG analysis (Fig. S7F). These terms were confirmed to be associated with aging (Fig. S2E; Fig. 3C), suggesting that parental age might have influenced the chronological age of the F_1_ generation 3-day-old flies used for RNA-seq. Taken together, our results demonstrated that parental age and photoperiod not only affected the transcriptomic profiles of the parents but also moderately affected the transcriptomic profiles of the F_1_ generation.

## Discussion

Despite increasing interest in the biological functions and regulatory mechanisms of mammalian m^6^A methylation, studies on this topic that use invertebrates as epitranscriptomic models are still scarce. *Drosophila* is an ideal epitranscriptomic model with a simplified m^6^A module system (23). In the past 5 years, studies related to *Drosophila* m^6^A have generally focused on the functional identification of m^6^A readers and writers with mammalian orthologs (10, 25–27). In the present study, we conducted a comprehensive investigation concentrating on the *Drosophila* transcriptomic, m^6^A epitranscriptomic, and metabolomic profiles affected by different durations of daily BLE. We attempted to understand the potential associations and interactions among m^6^A-related genes, 5′ UTR-enriched m^6^A epitranscriptomic regulation, aging-related genes, and the aging phenotype. Our results facilitated a systematic assessment of neuronal functions and implications of 5′ UTR-enriched m^6^A epitranscriptomic reprogramming induced by BLE and aging in *Drosophila*.

DNA methylation-based epigenetic clocks have been developed as a reliable tool for predicting mammalian chronological age (32). Epigenetic clock theory views aging as the unintended consequence of both developmental and maintenance programs and regards DNA methylation as the molecular footprint of this consequence (32). Although our study revealed limited changes in overall mRNA m^6^A levels between the 10-day-old and 25-day-old flies, the patterns of their m^6^A epitranscriptomic profiles were quite different and showed strong age specificity (Fig. 5A and B; Fig. S5A). Therefore, RNA methylation might also serve as a molecular estimator of chronological age, since epigenomic alterations are the essence of aging. Given the core position of mRNA in the central dogma, it could be modulated by posttranscriptional modifications in a more precise manner and further affect downstream translation and protein expression. With the rapid development of RNA modification sequencing technology at single-base resolution, RNA methylation-based epigenetic clocks might be further validated and applied.

Blue light has a greater tendency than other light colors to affect us through the disruption of our circadian physiology. Previous studies have demonstrated that daily blue light exposure can affect various phenotypes of *Drosophila*, including lifespan, neurodegeneration, mitochondria physiology, energy metabolism, and neurotransmitter levels (2, 28–30). Similar to previous findings, the main impacts of blue light on *Drosophila* observed in our study were related to neuronal functions, including aging and circadian rhythms. In our study, BLE indeed impacted the circadian rhythms using dark as the control; our findings can be supported by previous reports. For *Drosophila*, the photoreceptor protein cryptochrome has been reported to respond to blue light and contribute to circadian rhythms; the rhodopsin proteins have also been involved in entrainment of the circadian clock and photoinactivation in response to blue light (33–35). The differences in overall mRNA m^6^A levels (Fig. 1D), m^6^A writer expression levels (Fig. S5C), significant m^6^A peak numbers (Fig. 4B), 5′ UTR-enriched m^6^A methylation levels of *per* (Fig. 5B), and relative protein expression levels of per between the BD and DD flies (Fig. 5F and G) all showed that m^6^A methylation was involved in the impacts of BLE on *Drosophila*. Another finding of this work was that long-term BLE caused greater reprogramming in the multiomics profiles between the BD25 and DD25 flies than short-term BLE between the BD10 and DD10 flies (Figs. 3A, 3D, 4A, and 5B). Furthermore, it was unexpectedly found that the 3′ UTR-enriched m^6^A methylation of *Tor* was specifically reduced in the BD25 flies (Fig. 5A), although this region was not the binding and active site of the MTC (Fig. 5C). Our results clearly revealed that m^6^A methylation mediated transcriptional regulation imposed by BLE. Thus, we should pay more attention to the potential influence and hazards of increasing cumulative BLE in humans.

In most cases, the genome-wide mapping of vertebrate m^6^A methylation has revealed an enrichment bias predominantly localized near stop codons in 3′ UTRs, usually with the consensus motif “RRACH” (R = G/A, H = U/A/C) (36–38). Our high-resolution m^6^A epitranscriptomic mapping results demonstrated that the m^6^A methylation of *Drosophila* mRNAs was highly 5′ UTR enriched and alterable during aging and under different durations of daily BLE (Figs. 4C, 4E, 5A, and 5B). In human cells, the 5′ UTR-enriched m^6^A methylation of mRNAs is linked to cellular stress states and represents an alternative to the 5′ cap, enabling a cap-independent mode of translation initiation (39). In mammals, 5′ UTR-enriched m^6^A methylation might only be complementary to 3′ UTR-enriched m^6^A methylation. However, 5′ UTRs were shown to be not only the main regions of *Drosophila* m^6^A enrichment but also the binding and active sites of the MTC. Thus, 5′ UTR-enriched m^6^A methylation in *Drosophila* is likely to play some similar functional regulatory roles to 3′ UTR-enriched m^6^A methylation in mammals, such as modulating the degradation or alternative splicing of mRNAs. Overall, our results clearly revealed the main changes of 5′ UTR-enriched m^6^A methylation imposed by BLE, while the regulatory role and potential mechanism for m^6^A changes in 3′ UTR regions of specific genes (e.g. *Tor*) under blue light conditions require future investigation. m^6^A methylation could result in diverse regulatory consequences. Since the global transcriptome and m^6^A epitranscriptome were proven to be reciprocally inhibited in this study (Fig. 4H and I), we found that the relative expression levels of aging-related and circadian rhythm-related genes and the encoded proteins were particularly susceptible to inhibition (Figs. 5E, 5F, 6B, 6C, 6D, and 6E), implying regulatory consequences of m^6^A methylation on the stress-sensing genes of specific cell types.

The Toll and Imd signaling pathways contribute to the immune response of *Drosophila* by modulating the expression of several antimicrobial peptides (AMPs). Excessive immune activation can cause intestinal immune dysfunction, which will ultimately promote the aging of the host. Therefore, we used six AMP-related genes as biomarkers to evaluate the aging phenotype of the fly samples used in this study, and the RT-qPCR results between the 10-day-old and 25-day-old flies (Fig. S5D) were in line with our expectations. On the other hand, we found that these biomarker genes were expressed at significantly lower levels in the BD10 flies (Fig. S5D). It has been reported that an increased microbial influence during the early life of *Drosophila* adults extends the lifespan, whereas during later life, it shortens the lifespan (40). This might be the reason for the increased mortality of BD flies subjected to daily BLE. In addition, aging is a major risk factor for neurodegeneration-related conditions, including Alzheimer's disease and Parkinson's disease; further efforts are required to study the interaction between epigenetic modifications and aging. Notably, *Drosophila w^1118^* strain was used in our blue light experiments; previous study has indicated that this *Drosophila* strain showed premature retinal degeneration (41), especially that acute short-term BLE can directly induce retinal degeneration in *Drosophila* photoreceptors (42). Therefore, the observed molecular and phenotypic changes in our study might be the results of retinal degeneration induced by blue light, whether the changes and phenotypes observed in other *Drosophila* strains require future investigations.

Aging had a major impact on the m^6^A epitranscriptomic profiles obtained in our study (Figs. 4A, 4B, 4E, 5A, and 5B). In addition to promoting degradation or regulating translation, another main function of m^6^A methylation affecting DMGs might be in the alternative splicing of pre-mRNAs. It has been proven that the MTC is required for the sex-dependent regulation of the alternative splicing of the sex determination factor sex-lethal (*Sxl*) in *Drosophila* (43). However, this phenomenon was not easy to identify or validate in the majority of the DMGs, since accurate transcript quantification could be affected by redundant transcript isoforms or the presence of transcript isoforms with similar lengths. Surprisingly, this phenomenon was easy to identify in *Sod2*, since only two transcripts with different 3′ UTR lengths (*Sod2-RA* and *Sod2-RB*) have been found for this gene (Fig. S5A). Our results showed that the quantification results of the two transcripts were negatively correlated between the transcriptome and m^6^A epitranscriptome (Fig. S5B), suggesting that potential alternative splicing events of this gene might be negatively regulated by m^6^A methylation and altered with increasing age. Provided that alterations in the alternative splicing of aging-related genes are responsible for the aging phenotype, antiaging therapy targeting the m^6^A sites in these genes might be feasible in the future. In addition, previous study in *Drosophila* showed that the circadian clock protein period could change with age (44), whether and how m^6^A methylation is associated with the changes of those aging-related proteins require future investigations.

In conclusion, we used the model organism *Drosophila melanogaster* to investigate the epitranscriptomic and metabolomic roles in BLE-induced phenotypes including circadian disruption, neurodegeneration, and aging. Our results revealed that both BLE and aging could induce transcriptomic, m^6^A epitranscriptomic, and metabolomic reprogramming in *Drosophila.* We also showed that 5′ UTR-enriched m^6^A methylation with strong age specificity and could be altered by BLE, resulting in specific alterations in gene expression regulation related to neuronal functions. Our study provides a systematic assessment of BLE-induced and aging-induced m^6^A epitranscriptomic reprogramming in an animal model; the findings from this work will bring attention to potential hazards of cumulative BLE in humans.

## Materials and methods

### Fly colonies and genetics

We collected the eggs oviposited by 3-day-old *D. melanogaster w^1118^* adults from colonies maintained under a photoperiodic cycle of 12 h white fluorescent light: 12 h darkness (LD) after eclosion. These eggs were grown on a standard medium (80 g cornmeal, 125 g sucrose, 16 g yeast, and 5 g agar/L) under the LD photoperiodic cycle until 1 day after eclosion. We then transferred the 1-day-old adults to an RGX250E climatic chamber (Taisite, Tianjin, China) under the BD photoperiodic cycle and DD, respectively (Fig. 1A). The photon flux density of blue light (ARKNOAH 165W LED Aquarium Light, peak λ = 460 nm) was set as 30–50 μmol·m^−2^·s^−1^ determined by a photosynthetic photon flux density sensor (#HPL200P, HOPOCOLOR, Zhejiang, China). Under this daily exposure dose, the survival rates of adult flies were moderately decreased (Fig. 1B and C), and motor impairment was observed. To prevent the growth and activities of newborn larvae from increasing additional mortality to the target adults, we replaced the media every 3 days and counted the number of dead adults within the old medium to estimate survival rates (Fig. 1B and C). A total of five types of omics data sets were obtained in this study (see “Results”), each of which included four groups (Fig. 1A; Table S1). For RNA-seq and MeRIP-seq, each group contained three biological replicates; for untargeted metabolome quantification, each group contained five biological replicates (Table S1).

To construct MTC RNAi strain or mutant in vivo, *D. melanogaster w^1118^* (*+*/*+*) was used as the wild-type control. Other stocks that were used included a Gal4 driver line of *Act5C-Gal4*/*CyO* (Stock No. TB00033) provided by the *Drosophila* Stock Center at Tsinghua University, and a TRiP line of *Mettl3^HMS06028^* (Stock No. 80448) and an *fl(2)d* point mutation line of *cn^1^ fl(2)d^2^ bw^1^*/*CyO* (Stock No. 36302) purchased from the Bloomington *Drosophila* Stock Center. With the help of CyO and Sb balancers for phenotypic identification, hybridization was conducted to obtain the recombinant strains *Act5C-Gal4*/*+*, *Act5C-Gal4*/*Mettl3^HMS06028^*, and *fl(2)d^2^*/*+*.

### Total RNA extraction

Total RNA was isolated and dissolved in RNase-free water after the samples in bulk had been homogenized in TRIzol AG RNAex Pro Reagent (#AG21102, Accurate Bioeng. Co., Ltd., Hunan, China) with glass beads using a homogenizer (#Tissuelyser-48, Jingxin Industrial Development Co., Ltd., Shanghai, China). The concentration of total RNA extracted from each replicate was measured at 260 nm on a NanoDrop 2000 Spectrophotometer (Thermo Scientific, Waltham, MA, USA).

### Construction of RNA-seq libraries

The poly(A)-RNA-seq libraries of adult male heads (BD10, BD25, DD10, and DD25 heads) and F_1_ generation whole adult male flies (BD8F_1_, BD23F_1_, DD8F_1_, and DD23F_1_ flies) were mainly prepared by Berry Genomics (Beijing, China). Briefly, the RNA concentration was quantified using a Qubit RNA HS Assay Kit (#Q32852, Invitrogen, Carlsbad, CA, USA) on a Qubit 2.0 Fluorometer (#Q32866, Invitrogen), and RNA integrity was evaluated using an Agilent RNA 6000 Nano Kit (#5067-1511, Agilent Technologies, CA, USA) on an Agilent Bioanalyzer 4200 TapeStation (Agilent Technologies) according to an RNA integrity number >8.0. Poly(A)-RNA was purified from the extracted total RNA using Dynabeads Oligo(dT)25 (#61002, Invitrogen). An AMPure XP system (#A63882, Beckman Coulter, Brea, CA, USA) was used for further purification, and fragments of ∼350 bp in length were selected. Strand-specific sequencing libraries were generated using an NEBNext Ultra RNA Library Prep Kit for Illumina (#E7530L, NEB, Ipswich, MA, USA) according to the manufacturer's protocols, including first-strand and second-strand cDNA synthesis, adaptor ligation, and library amplification with index sequences.

### Construction of MeRIP-seq libraries

We prepared poly(A)-MeRIP-seq libraries from whole adult male flies (BD10, BD25, DD10, and DD25 flies). Briefly, the extracted total RNA was subjected to poly(A)-selection using Oligo d(T)_25_ Magnetic Beads (#S1419S, NEB). Purified poly(A)-RNA was fragmented into ∼100 nt fragments for the construction of both m^6^A IP and input MeRIP-seq libraries. In addition, the poly(A)-RNA was used for determining m^6^A levels by LC-MS/MS after enzymatic hydrolysis into ribonucleosides and for further RT-qPCR validation without fragmentation. m^6^A IP was conducted using an EpiMark N6-Methyladenosine Enrichment Kit (#E1610S, NEB) following the product manual. The resultant m^6^A-enriched and untreated poly(A)-RNA fragments were then used to construct strand-specific IP and input libraries for MeRIP-seq, respectively. VAHTS Universal V6 RNA-seq Library Prep Kit for Illumina (#NR604-02, Vazyme Biotech. Co., Ltd., Jiangsu, China) and VAHTS RNA Adapters set3 for Illumina (#N809, Vazyme Biotech. Co., Ltd.) provided the necessary reagents for the fragmentation of poly(A)-RNA, first-strand and second-strand cDNA synthesis, adaptor ligation, and library amplification with index sequences.

### RNA-seq and MeRIP-seq

Berry Genomics carried out RNA-seq and MeRIP-seq of the prepared libraries on a NovaSeq 6000 platform (Illumina, San Diego, CA, USA). Library concentrations and quality were finally assessed on the Agilent Bioanalyzer 4200 TapeStation before loading onto the sequenator. In 2 × 150 bp paired-end (PE150) sequencing mode, a total of 48 libraries were sequenced, and each library generated ∼31.5–138.6 million raw reads (4.7–20.8 Gb; Table S1).

### LC-MS/MS quantification of the untargeted metabolome

Beijing Genomics Institution (BGI, Guangdong, China) performed the extraction and LC-MS/MS quantification of the untargeted metabolome of whole adult male flies (BD10, BD25, DD10, and DD25 flies). Briefly, following a previously published method (45), ∼8 mg of the fly samples from each replicate were extracted by directly adding 800 μL of precooled extraction reagent (methanol: acetonitrile: water = 2:2:1). Internal standards were also added for quality control (QC) during sample preparation. The samples were homogenized using a homogenizer (#JXFSTPRP, Jingxin Industrial Development Co., Ltd.), sonicated for 10 min, incubated at −20°C for 1 h, and centrifuged at 25,000 rpm at 4°C for 15 min. Finally, the supernatants and QC samples of the same volume were transferred to autosampler vials for LC-MS/MS analysis. To improve compound coverage, an ultra-performance liquid chromatography (#Waters 2D UPLC, MA, USA) tandem high-resolution mass spectrometer (#Q Exactive HF, Thermo Scientific) was used for separating and detecting the compounds in both positive and negative ion modes.

The output raw LC-MS/MS data were imported into Compound Discoverer 3.1 (Thermo Scientific), and the combined results of compound identification based on the BGI Library (BGI in-house-developed standard library), mzCloud, and ChemSpider (HMDB, KEGG, and LipidMaps) databases and corresponding peak areas were exported. These exported results were subjected to further data processing, including the normalization of the peak areas to obtain the relative peak areas using probabilistic quotient normalization (46), the correction of the batch effect using QC-based robust locally estimated scatterplot smoothing (LOESS) signal correction (45), and the filtration of the identified compounds based on the coefficient of variation of the relative peak areas in all QC samples.

### Analyses of RNA-seq data

Adaptors and raw reads containing low-quality bases were removed by applying fastp (version 0.20.1) (47) to all RNA-seq raw data, including the MeRIP-seq input libraries. The resulting reads of at least 50 bp were mapped to the reference genome (FB2021_02) released by the FlyBase database (http://flybase.org/; 48). The featureCounts program (49) within the SourceForge Subread package (version 2.0.2) was used to count the reads that were mapped to genes. The genes that showed an average read count of less than 10 in any group or lacked read count in any replicate were primarily filtered. The R package DESeq2 (version 1.30.0; 50) was used for the identification of DEGs by setting a false discovery rate (FDR)-corrected *P*-value <0.05 and fold change ≥2 as the thresholds for significance (Table S2) and used for read count normalization across samples by size factors. Gene expression was measured based on normalized read counts in this study. Transcripts were quantified according to fragments per kilobase million values using Cufflinks (version 1.30.0; 51). We performed t-SNE dimension reduction analyses using the R (version 4.0.3; R Core Team 2020) package Rtsne (version 0.15; https://github.com/jkrijthe/Rtsne), displayed gene expression in heatmaps using the R package pheatmap (version 1.0.12; https://CRAN.R-project.org/package=pheatmap), illustrated common and unique DEGs in Venn diagrams using the R package VennDiagram (version 1.6.20; https://CRAN.R-project.org/package=VennDiagram), and generated a volcano plot using the R package ggplot2 (version 3.3.3; https://ggplot2.tidyverse.org) for the comparison of the RNA-seq data between BD25 and DD25 fly heads. GO and KEGG enrichment analyses of the DEGs were conducted by using either Metascape (version 3.5; https://metascape.org/) or the R package clusterProfiler (version 3.18.1). A PPI network based on the DEGs identified in the single-factor comparisons between groups was also generated via Metascape. GSEA software (version 4.2.0; 52) was used to determine the statistical significance of the priori-defined gene sets. The R package ggstatsplot (version 0.9.1) was used to visualize scatter diagram.

### Analyses of MeRIP-seq data

We primarily processed all the raw data of the MeRIP-seq IP libraries with the same upstream pipeline employed for RNA-seq. The mapping results of both the MeRIP-seq IP and input libraries were provided for calling the m^6^A peaks of each replicate and group and the differential m^6^A peaks of the single-factor comparisons between groups using the R package exomePeak2 (version 0.99.92; https://bioconductor.org/packages/release/bioc/html/exomePeak2.html), with a Poisson generalized linear model as the quantitative method (Table S3). The “consistent_peak” option was used if applicable. To reduce false positives, the called m^6^A peaks were regarded as significant and retained for subsequent analyses according to the following thresholds: sequencing coverage of the input library ≥50 reads, peak width ≤1500 bp, fold change ≥2, *P* < 0.01, and FDR < 0.01 (Table S3). Similarly, the called differential m^6^A peaks were regarded as significant under the following thresholds: sequencing coverage of both the IP and input libraries ≥50 reads, peak width ≤1500 bp, fold change ≥1.5, *P* < 0.01, and FDR < 0.01 (Table S3). HOMER (version 4.11; 53) was used for de novo motif searching around peak summit-centered 250 bp regions. The R packages ChIPseeker (version 1.18.0; 54) and Guitar (version 2.8.0; 55) were used for the annotation and representation of the distribution characteristics of the significant (differential) m^6^A peaks. We conducted t-SNE analyses of the IP data at both the gene level (based on gene expression) and the peak level (based on the normalized read counts of the IP/input data sets). IGV software (version 2.11.9; 56) was used to display the read coverage tracks of target genes using the mapping results of both the IP and input libraries in bigWig format. To confirm the binding and active sites of the MTC among the genes of interest, we used the MeRIP-seq data deposited in the NCBI Gene Expression Omnibus (GEO) database (https://www.ncbi.nlm.nih.gov/geo/) under accession number GSE155662 (26) for the further examination of IGV tracks, which derived from whole 1-day-old to 2-day-old adult flies including the following *Drosophila* strains: *yw* (males and females), *Mettl3^SK2^/Df* (males), *Mettl14^SK1^* (males), and *Hakai^SH4^* (males).

### Analyses of metabolome data

The identified compounds were annotated with different confidence levels (levels 1 to 5) by BGI. A total of 208 compounds (126 and 82 identified in positive and negative ion modes, respectively) within the relatively high confidence levels (levels 1 to 3) were selected for subsequent analyses in this study. According to the metabolite identification credibility evaluation criteria specified by BGI, level 1 indicated the compounds that could be accurately determined based on the standard database and experimental data (MS2 spectrum score ≥60 and retention time [RT] deviation ≤0.2 min); level 2 indicated the compounds whose structural formulas matched the standards database (no RT value or RT deviation >0.2 min); and level 3 indicated the compounds with structural regions that could be matched with the standards database but needed further verification (MS2 spectrum score <60). The normalized relative peak areas of the 208 compounds were used for PCA and heatmap visualization (top 50 high-quantified compounds) with clustering analysis results conducted with MetaboAnalyst (version 5.0, https://www.metaboanalyst.ca/; 57). Differential compounds were identified in the single-factor comparisons between groups by applying Student's t test (Table S2). These differential compound sets were represented in a Venn diagram using EVenn (http://www.ehbio.com/test/venn; 58) and used for KEGG enrichment analyses performed with MetaboAnalyst (57).

### LC-MS/MS quantification of m^6^A levels

We digested 150 ng of poly(A)-RNA prepared from whole adult male flies (BD10, BD25, DD10, and DD25 flies) via the aforementioned procedure with 5 μL buffer containing 2 U nuclease P1, 250 mM NaCl, and 25 mM ZnCl_2_ at 37°C for 2 h and then added 2 U FastAP Thermosensitive Alkaline Phosphatase (#EF0651, Thermo Scientific) and 3.5 μL of 10× FastAP Buffer, followed by another 4 h of incubation at 37°C. The resulting digested nucleoside mixture was used for LC-MS/MS quantification in the School of Life Sciences at Peking University. Briefly, 5 μL of each digested sample was injected into an ^18^C reversed-phase column coupled online to an Agilent K6460 Triple Quad Mass Spectrometer (Agilent Technologies). In multiple reaction monitoring positive electrospray ionization mode, adenosine and m^6^A were quantified based on the transition of the parent ribonucleoside to the deglycosylated base ion (268.1–136.1 for adenosine, 282.1–150.1 for m^6^A). Finally, we obtained the absolute quantities of adenosine and m^6^A using external calibration curves derived from adenosine (#A9251, Sigma-Aldrich, St. Louis, MO, USA) and m^6^A standards (#S3190, Selleck Chemicals, Houston, TX, USA), respectively.

### Cell lines and culture

Embryonic-derived *Drosophila* S2 cells were grown in InsectPro Sf9 Insect Cell Serum-Free Medium (#H832KJ, Basalmedia Biotech. Co., Ltd., Shanghai, China) supplemented with 10% FBS (#12103C, Sigma-Aldrich) at 27°C.

### RNAi

Following the manufacturer's protocol, the T7 RiboMAX Express RNAi System (#P1700, Promega, WI, USA) was used for the synthesis of double-stranded (dsRNA) molecules. We also prepared dsRNA targeting green fluorescent protein (GFP) as the control for all other RNAi experiments in vitro. A total of 20 μg dsRNA targeting *Mettl3*, *fl(2)d*, or *Tor* and 10 μL FuGENE HD Transfection Reagent (#E2311, Promega) were added to 100 μL serum-supplemented medium containing cultured S2 cells at a density of 10^6^ cells/mL. After 48 h of incubation, we removed the remaining medium and collected the S2 cells for subsequent experiments.

### RT-qPCR

Following the manufacturer's instructions, we used a HiFiScript gDNA Removal cDNA Synthesis Kit (#CW2582M, CWBIO, Jiangsu, China) to synthesize first-strand cDNA from 150 ng poly(A)-RNA prepared from whole adult male flies (BD10, BD25, DD10, and DD25 flies) via the aforementioned procedure or 1 μg total RNA extracted from the constructed RNAi S2 cells. Then, the cDNA products were used for RT-qPCR after mixing with MagicSYBR Mixture (#CW3008M, CWBIO). The targeted genes of interest in this study included m^6^A-related, aging-related, and circadian rhythm-related genes and potential aging biomarkers of *Drosophila* (Table S4; 59). The applied primer pairs (Table S4) were all designed using Primer-BLAST. We used ribosomal protein 49 (*Rp49*) as the internal control gene and the DD10 flies or GFP as the calibrator(s). The relative expression levels of target mRNAs were calculated using the 2^−ΔΔCt^ method.

### Western blotting

In this study, ∼15 mg samples of whole adult male flies or 10^6^ S2 cells from each replicate were homogenized in 150 μL RIPA Lysis Buffer (#P0013B, Beyotime Biotech. Co., Ltd., Shanghai, China) with freshly added 1 mM PMSF (#ST506, Beyotime Biotech Co., Ltd.). The resulting suspension was centrifuged at 13,000 rpm at 4°C for 30 min, and the supernatant was subsequently collected for Western blotting. Bicinchoninic acid (BCA) assays were performed using a BCA Protein Quantification Kit (#20201ES76, Yeasen Biotech Co., Ltd., Shanghai, China) to measure the protein concentration. The samples were subjected to sodium dodecyl sulfate-polyacrylamide gel electrophoresis, transferred to a polyvinylidene fluoride membrane, and subsequently probed with a primary antibody and washed. The primary antibodies used for Western blotting included a β-Actin Mouse Monoclonal Antibody (#AF5001, Beyotime Biotech Co., Ltd.), METTL3 Polyclonal antibody (#15073-1-AP, Proteintech, USA), METTL14 Polyclonal antibody (#HPA038002, Sigma, USA), WTAP Monoclonal antibody (#60188-1-Ig, Proteintech), Recombinant Anti-mTOR antibody [EPR390(N)] (#ab134903, Abcam, England), and Recombinant Anti-PER3 antibody (EPR13038) (#ab177482, Abcam, USA). After incubation with the corresponding secondary antibody, each targeted protein was detected using Clarity Western ECL Substrate (#1705060, Bio-Rad, Hercules, CA, USA) and photographed with a Tanon 5200 Chemiluminescent Imaging System (Tanon, Shanghai, China). Western blotting images were quantified using the integrated density values (IDVs) measured by ImageJ software (version 1.53e).

### Immunofluorescence

The midguts of adults of the *Act5C-Gal4*/*+*, *Act5C-Gal4*/*Mettl3^HMS06028^*, and *fl(2)d^2^*/*+* recombinant *Drosophila* strains were dissected, fixed in PBS containing 4% formaldehyde and 2% Triton X-100 (#CT104200-500A, Aqueous, CA, USA) for 40 min, washed with PBT buffer (1× PBS containing 0.1% BSA and 0.1% Triton X-100), incubated with an m^6^A primary antibody (#202 011, SYSY, Goettingen, Germany) at 4°C overnight, and then incubated with a Goat anti-Rabbit IgG (H + L) Cross-Adsorbed Secondary Antibody, Alexa Fluor 488 (#A-11008, Invitrogen) for 1 h. Tissues were mounted in PBT buffer and stained with DAPI Staining Solution (#ab228549, Abcam). A FLUOVIEW FV3000 confocal laser scanning microscope (Olympus, Shinjuku, Japan) was used to capture immunofluorescence images of the samples.

### Survival test

Briefly, we transferred eclosion adults to a climatic chamber under continuous BLE (30–50 μmol·m^−2^·s^−1^) and supplied with customized medium. The methylation inhibitor DAA (#GC14713, Glpbio, Montclair, CA, USA), or the mTOR inhibitor rapamycin (#HY-10219, MedChemExpress, Monmouth Junction, NJ, USA), was added to *Drosophila* medium with a final concentration of 30 μM or 200 μM, respectively. We replaced the medium every 3 days and counted the number of dead adults to estimate survival rates. After the last count on the 18th day, the remaining live adults were counted and sampled for Western blot experiments.

### Statistics

Experiments were performed in at least three replicates, and representative data were shown. Statistical data were presented as the mean ± SD. The significance of the survival rates between the BD and DD flies was measured with the log-rank test. The statistical significance of single-factor and two-factor analyses was determined with the unpaired, two-tailed Student's t tests and two-way ANOVA, respectively. In all analyses, *P < 0.05, **P < 0.01, ***P < 0.001, ****P < 0.0001.

## Supplementary Material

pgad390_Supplementary_Data

## Data Availability

The raw and processed data of this study have been deposited in the NCBI GEO database under the accession number GSE197397. Specifically, the MeRIP-seq (including m^6^A IP and input) data of whole adult male flies, RNA-seq data of adult male heads, and RNA-seq data of the F_1_ generation whole adult male flies have been deposited under accession numbers GSE197395, GSE197370, and GSE197382, respectively.
