# Supplementary material for: Systematic assessment of transcriptomic and metabolic reprogramming by blue light exposure coupled with aging
Source: PNAS Nexus. 2023 Dec 5;2(12):pgad390. doi: 10.1093/pnasnexus/pgad390 (PMC10697416; doi:10.1093/pnasnexus/pgad390)
Supplement: pgad390_Supplementary_Data [file pgad390_supplementary_data.zip › PNASNEXUS-PNASNEXUS-2023-00402R-s01.pdf]

Supplemental Figures and Tables

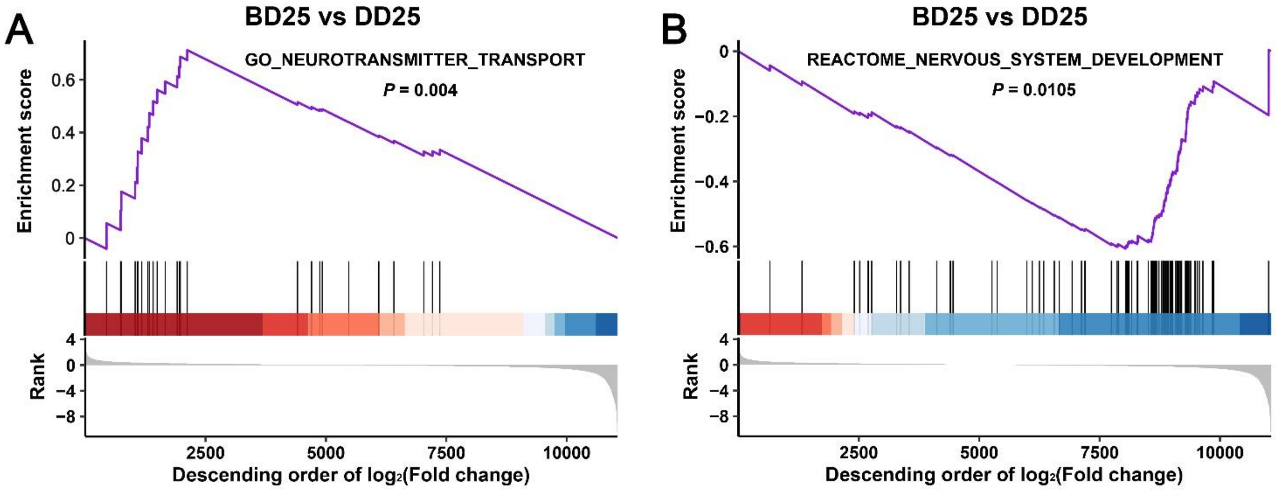

Figure S1. Gene set enrichment analyses (GSEAs) of the polyadenylated (poly-A) RNA sequencing (RNA-seq) data between the BD25 and DD25 *Drosophila melanogaster* w^{1118} adult male heads provide additional evidence that blue light exposure (BLE) influences the cephalic nervous system. BD25 and DD25: 25-day-old adult flies reared under a photoperiodic cycle of 12 h low-intensity BLE: 12 h darkness (BD) and constant darkness (DD), respectively. (**A**) GSEA results of the GO_NEUROTRANSMITTER_TRANSPORT. (**B**) GSEA results of REACTOME_NERVOUS_SYSTEM_DEVELOPMENT.

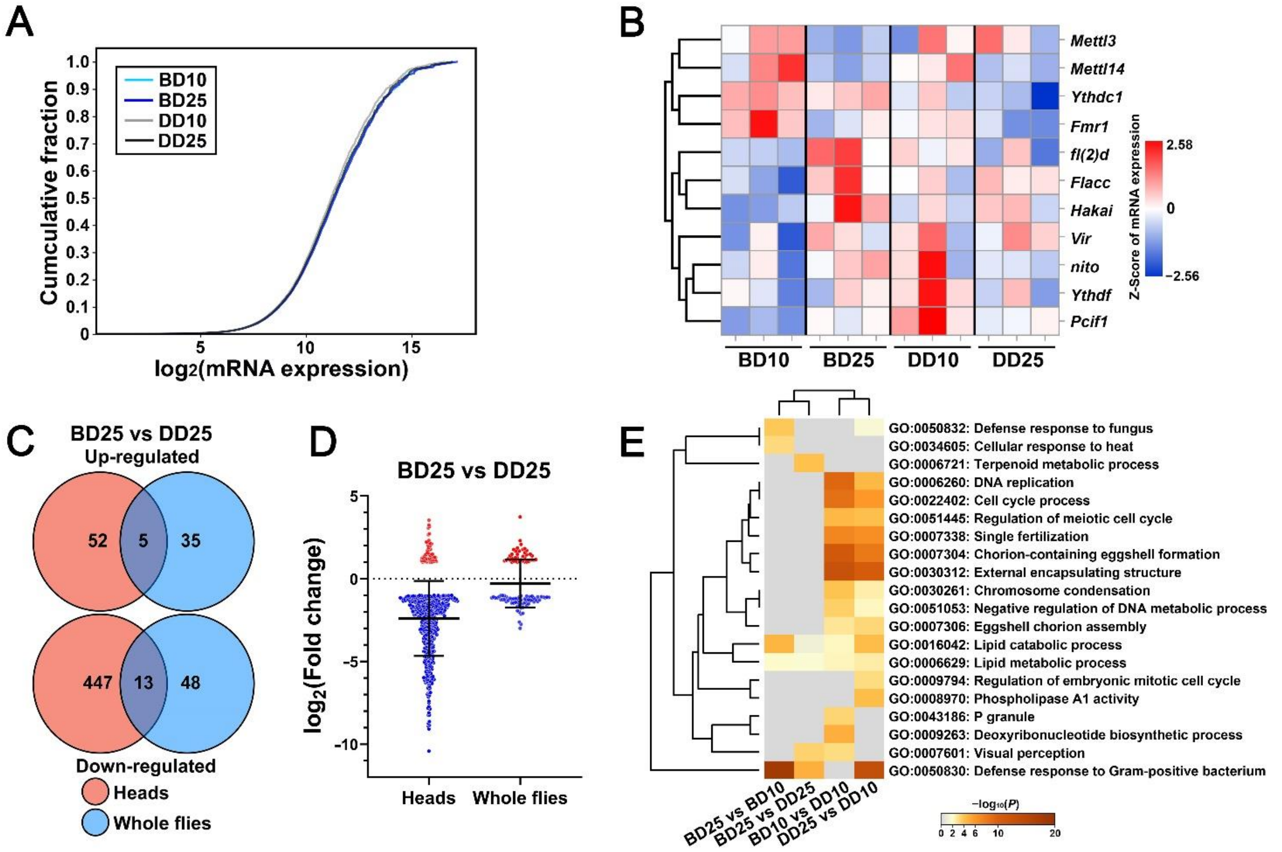

Figure S2. Supplementary results for the poly-A RNA sequencing (RNA-seq) of whole adult male flies show transcriptomic reprogramming induced by BLE and aging. BD10 and DD10: 10-day-old adult flies reared under the photoperiodic cycle of BD and DD, respectively. (**A**) Cumulative distribution of poly-A RNA expression. (**B**) Heatmap and clustering analysis of m^6A- and N^6,2'-O-dimethyladenosine (m^6Am)-related genes. (**C**) Venn diagrams displaying the common and unique up- and downregulated differentially expressed genes (DEGs) (BD25 versus DD25 flies) between the adult male heads and whole flies. A false discovery rate-corrected P-value (FDR) < 0.05 and fold change ≥ 2 were used as criteria for identifying DEGs. (**D**) Scatter plot showing the expression levels differences of the DEGs (BD25 versus DD25 flies) between adult male heads and whole flies. (**E**) Enrichment and clustering analyses showing the top 20 Gene Ontology (GO) terms of the DEGs identified in the single-factor comparisons between groups.

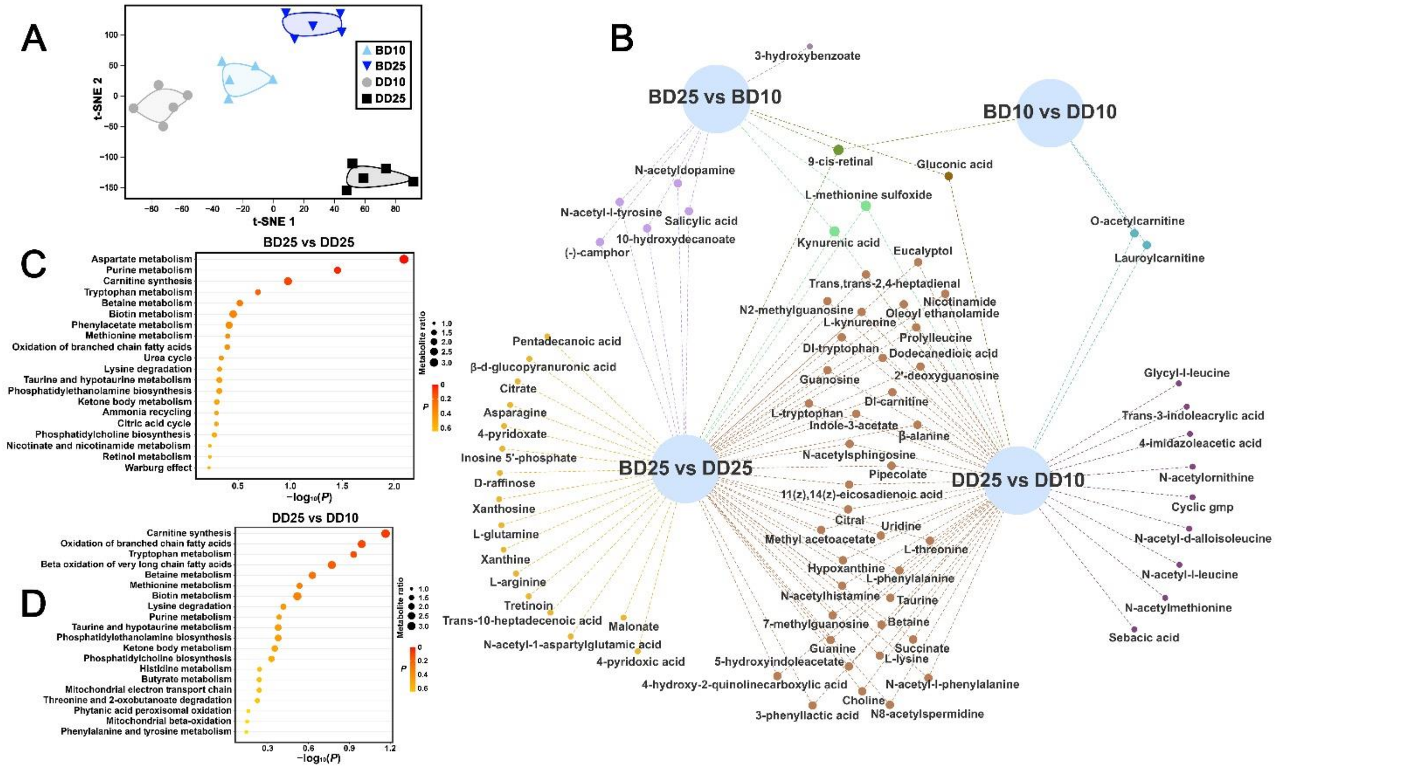

Figure S3. Supplementary results for the untargeted metabolome of whole adult male flies show metabolomic reprogramming induced by BLE and aging. (**A**) t-SNE dimension reduction analysis based on 208 quantified compounds. (**B**) Venn diagram displaying the common and unique differential compounds identified in the single-factor comparisons between groups. $P < 0.05$ and a fold change ≥ 2 were used as the criteria for identifying differential compounds. (**C**) Enrichment analyses showing the top 20 Kyoto Encyclopedia of Genes and Genomes (KEGG) terms between the BD25 and DD25 flies based on 63 differential compounds. (**D**) Enrichment analyses showing the top 20 Kyoto Encyclopedia of Genes and Genomes (KEGG) terms between the DD25 and DD10 flies based on 53 differential compounds.

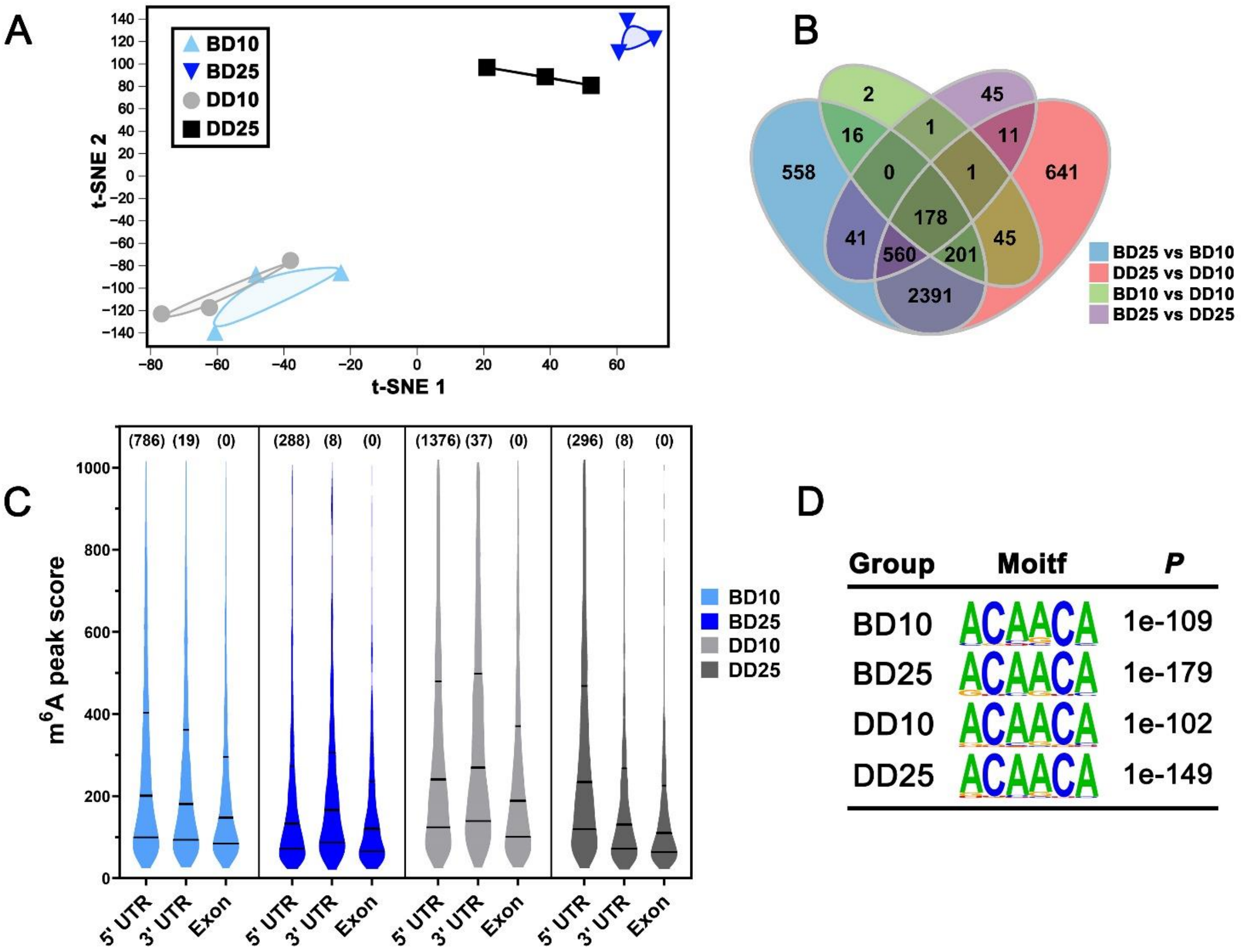

Figure S4. Supplementary results for the poly-A methylated RNA immunoprecipitation and sequencing (MeRIP-seq) of whole adult male flies indicate that BLE and aging induce m^{6}A epitranscriptomic reprogramming. (**A**) t-SNE dimension reduction analysis based on the enrichment levels (IP/input) of 6,332 consistent m^{6}A peaks for all fly samples. (**B**) Venn diagram displaying the common and unique differentially methylated genes (DMGs) identified in the single-factor comparisons between groups. (**C**) m^{6}A peak enrichment levels measured by peak scores [log$_2$(*P*-value)] across different annotated locations in genes. The numbers at the top of the panel indicate the significantly enriched m^{6}A peaks with a peak score > 1,024 (2^{10}) defined as "infinite" by exomePeak2. A fold change ≥ 2 (1.5), *P* < 0.01, and an FDR < 0.01 were used as criteria for identifying significant (differential) m^{6}A peaks. (**D**) "ACAACA" is one of the most frequent and significant motifs identified by HOMER based on the significant m^{6}A peaks of the examined groups.

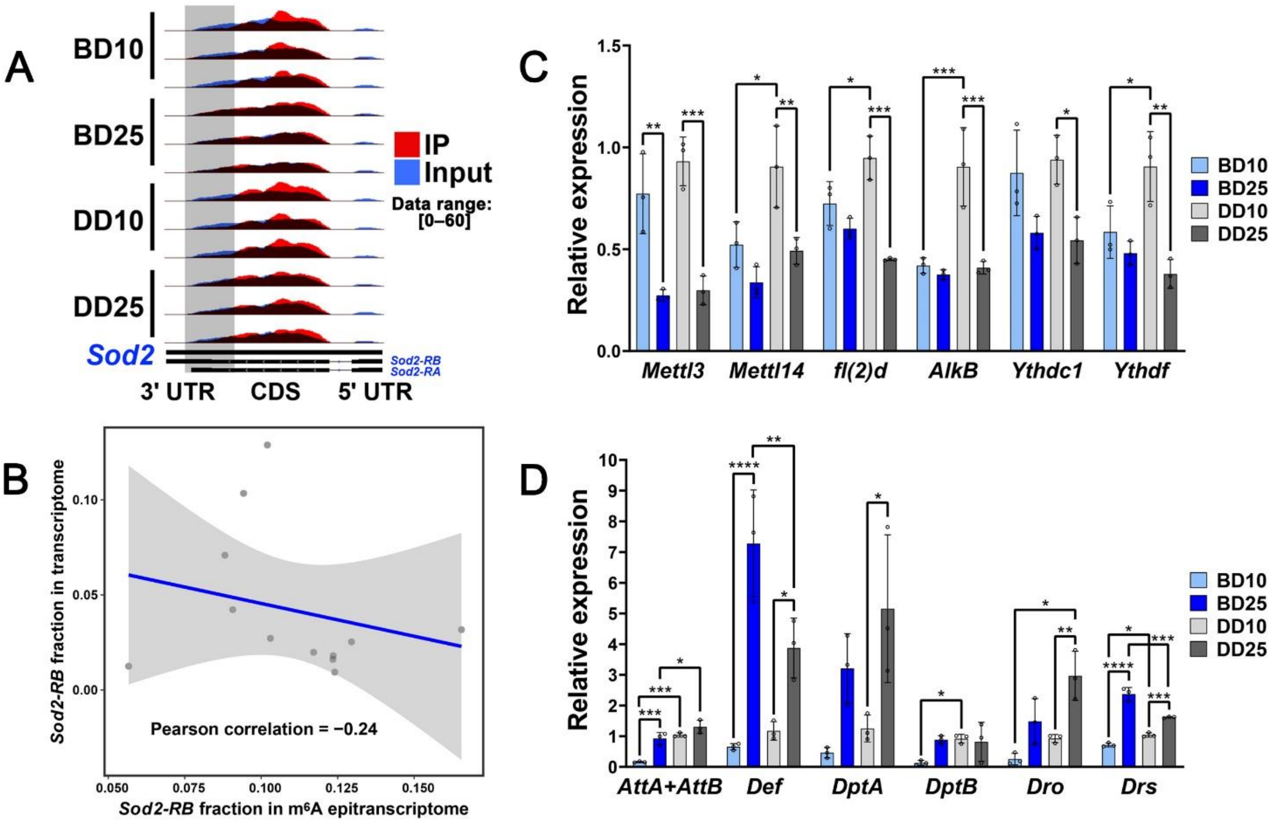

Figure S5. m^6A epitranscriptomic reprogramming induced by aging and BLE impacts the alternative splicing of *Sod2*, with additional RT-qPCR validation of m^6A-related mRNAs and potential aging biomarkers. (**A**) Integrative Genomics Viewer (IGV) tracks displaying the differential read coverage across *Sod2* according to the MeRIP-seq IP and input data of whole adult male flies. The data ranges of all the IGV tracks in each panel have been scaled to the same level. (**B**) Scatter diagram showing that the *Sod2-RB* transcript quantification results are negatively correlated between the IP and input data. (**C**) RT-qPCR results of m^6A-related mRNAs in whole adult male flies. (**D**) RT-qPCR results of potential aging biomarkers in whole adult male flies. Mean ± SD; (*) $P < 0.05$, (**) $P < 0.01$, (***) $P < 0.001$, (****) $P < 0.0001$, measured with two-way ANOVA.

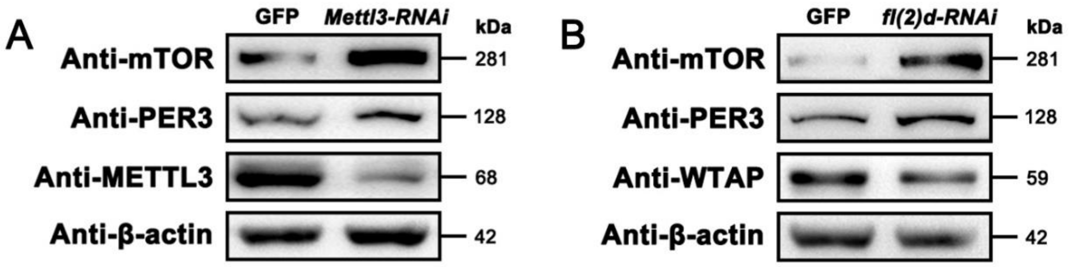

Figure S6. Western blot validation of the methyltransferase complex (MTC) RNA interference (RNAi) S2 cells implies correlations among Mettl3, fl(2)d, Tor, and per at the relative protein expression level. (**A**) Western blotting of Tor, per, and Mettl3 between the green fluorescent protein (GFP) control and *Mettl3*-RNAi S2 cells. (**B**) Western blotting of Tor, per, and fl(2)d between the GFP control and *fl(2)d*-RNAi S2 cells.

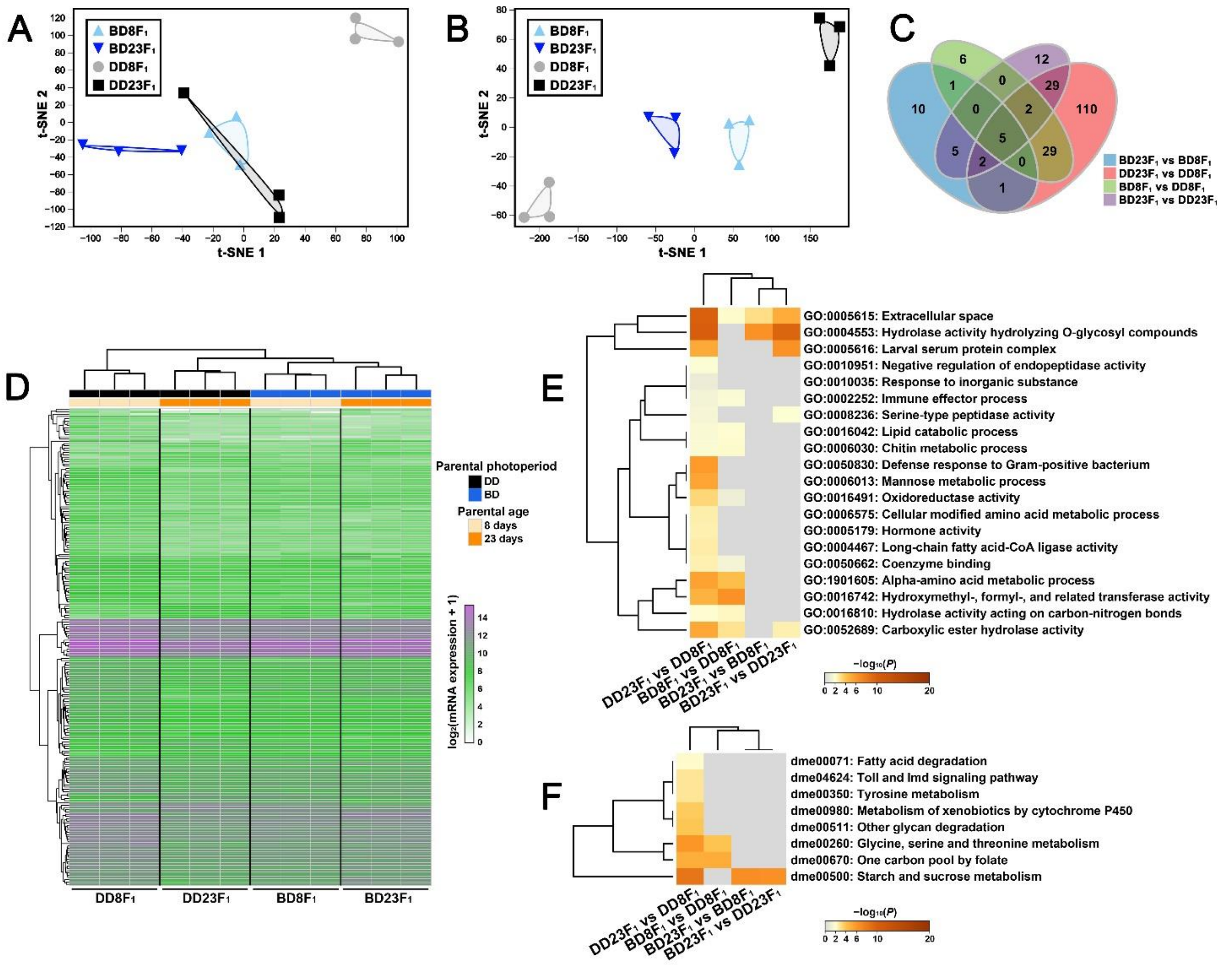

Figure S7. Poly-A RNA-seq of the F_1 generation whole adult male flies shows slight transcriptomic reprogramming induced by the influence of parental photoperiod and aging. BD8F_1 and BD23F_1: 3-day-old adult flies grown from eggs of the 8- and 23-day-old parents reared under the BD photoperiodic cycle. DD8F_1 and DD23F_1: 3-day-old adult flies grown from the eggs of 8- and 23-day-old parents reared under DD. (**A**) t-SNE dimension reduction analysis based on 13,848 genes identified in the single-factor comparisons between groups. (**B**) t-SNE dimension reduction analysis based on 211 DEGs identified in the single-factor comparisons between groups. An FDR < 0.05 and a fold change ≥ 2 were used as criteria for identifying DEGs. (**C**) Venn diagram displaying the common and unique DEGs identified in the single-factor comparisons between groups. (**D**) Heatmap and clustering analysis of the 211 DEGs identified in the single-factor comparisons between groups. (**E**) Enrichment and clustering analyses showing the top 20 GO based on the DEGs identified in the single-factor comparisons between groups. (**F**) Enrichment and clustering analyses showing the top 20 KEGG terms based on the DEGs identified in the single-factor comparisons between groups.

Supplemental table legends

Table S1. The omics techniques and treatments employed in this study, with the sequencing read counts and mapping results of the samples used.

Table S2. The differentially expressed genes (DEGs), differential m^6A peaks, or differential compounds identified in the single-factor comparisons between groups for each omics data set.

Table S3. The significant m^6A peaks of the groups and differential m^6A peaks identified in the single-factor comparisons between groups based on the methylated RNA immunoprecipitation and sequencing (MeRIP-seq) data of w^{1118} whole adult male flies.

Table S4. The RT-qPCR primer pairs designed and used in this study.
